# Miconazole Promotes Cooperative Ability of a Mouse Model of Alzheimer Disease

**DOI:** 10.1093/ijnp/pyac061

**Published:** 2022-09-16

**Authors:** Ze Wang, Yanli Zhang, Weixi Feng, Yingting Pang, Sijia Chen, Shixin Ding, Yan Chen, Chengyu Sheng, Charles Marshall, Jingping Shi, Ming Xiao

**Affiliations:** Jiangsu Province Key Laboratory of Neurodegeneration, Center for Global Health, Nanjing Medical University, Nanjing, China; Brain Institute, the Affiliated Nanjing Brain Hospital of Nanjing Medical University, Nanjing, China; Jiangsu Province Key Laboratory of Neurodegeneration, Center for Global Health, Nanjing Medical University, Nanjing, China; Jiangsu Province Key Laboratory of Neurodegeneration, Center for Global Health, Nanjing Medical University, Nanjing, China; Brain Institute, the Affiliated Nanjing Brain Hospital of Nanjing Medical University, Nanjing, China; Jiangsu Province Key Laboratory of Neurodegeneration, Center for Global Health, Nanjing Medical University, Nanjing, China; Brain Institute, the Affiliated Nanjing Brain Hospital of Nanjing Medical University, Nanjing, China; Jiangsu Province Key Laboratory of Neurodegeneration, Center for Global Health, Nanjing Medical University, Nanjing, China; Brain Institute, the Affiliated Nanjing Brain Hospital of Nanjing Medical University, Nanjing, China; Jiangsu Province Key Laboratory of Neurodegeneration, Center for Global Health, Nanjing Medical University, Nanjing, China; Brain Institute, the Affiliated Nanjing Brain Hospital of Nanjing Medical University, Nanjing, China; Jiangsu Province Key Laboratory of Neurodegeneration, Center for Global Health, Nanjing Medical University, Nanjing, China; Brain Institute, the Affiliated Nanjing Brain Hospital of Nanjing Medical University, Nanjing, China; Jiangsu Province Key Laboratory of Neurodegeneration, Center for Global Health, Nanjing Medical University, Nanjing, China; Department of Rehabilitation Sciences, University of Kentucky Center of Excellence in Rural Health, Hazard, USA; Brain Institute, the Affiliated Nanjing Brain Hospital of Nanjing Medical University, Nanjing, China; Department of Neurology, the Affiliated Nanjing Brain Hospital of Nanjing Medical University, Nanjing, China; Jiangsu Province Key Laboratory of Neurodegeneration, Center for Global Health, Nanjing Medical University, Nanjing, China; Brain Institute, the Affiliated Nanjing Brain Hospital of Nanjing Medical University, Nanjing, China

**Keywords:** Alzheimer disease, cooperative behavior, miconazole, myelin sheath, oligodendrocytes

## Abstract

**Background:**

Cooperative defect is 1 of the earliest manifestations of disease patients with Alzheimer disease (AD) exhibit, but the underlying mechanism remains unclear.

**Methods:**

We evaluated the cooperative function of APP/PS1 transgenic AD model mice at ages 2, 5, and 8 months by using a cooperative drinking task. We examined neuropathologic changes in the medial prefrontal cortex (mPFC). Another experiment was designed to observe whether miconazole, which has a repairing effect on myelin sheath, could promote the cooperative ability of APP/PS1 mice in the early AD-like stage. We also investigated the protective effects of miconazole on cultured mouse cortical oligodendrocytes exposed to human amyloid β peptide (Aβ_1-42_).

**Results:**

We observed an age-dependent impairment of cooperative water drinking behavior in APP/PS1 mice. The AD mice with cooperative dysfunction showed decreases in myelin sheath thickness, oligodendrocyte nuclear heterochromatin percentage, and myelin basic protein expression levels in the mPFC. The cooperative ability was significantly improved in APP/PS1 mice treated with miconazole. Miconazole treatment increased oligodendrocyte maturation and myelin sheath thickness without reducing Aβ plaque deposition, reactive gliosis, and inflammatory factor levels in the mPFC. Miconazole also protected cultured oligodendrocytes from the toxicity of Aβ_1-42_.

**Conclusions:**

These results demonstrate that mPFC hypomyelination is involved in the cooperative deficits of APP/PS1 mice. Improving myelination through miconazole therapy may offer a potential therapeutic approach for early intervention in AD.

**Significance Statement**

Cooperative defect is one of the earliest manifestations of patients with Alzheimer’s disease (AD). Exploring the underlying mechanism would assist in the early prevention and treatment of AD. Recently, our group developed a cooperative drinking system that is able to quantitatively analyze cooperation willingness and efficiency of mice. In this study, by use of this behavioral paradigm, we characterized age-dependent deficits in the cooperative ability of APP/PS1 transgenic AD mice, which was associated with hypomyelination in the medial prefrontal cortex (mPFC), a key regulatory region of social behaviors. We also found that miconazole treatment improved cooperative function and mPFC myelination of this AD mouse model. To the best of our knowledge, it is the first report on the social cooperative alterations of AD model mice, its neuropathological mechanism and possible treatment strategies.

## INTRODUCTION

*Social cooperation* refers to the way in which individuals collaborate to achieve goals or maximize benefits (Henry et al., 2016; Tomasello and Vaish, 2013). A progressive decline in cooperative ability occurs during Alzheimer disease (AD) progression (Mohs et al., 2000; Rankin et al., 2008). Notably, patients with mild cognitive impairment are able to participate in general social activities, showing only egotistic tendencies, but appear apathetic and irritable, with other distressing personality changes during “altruistic” and “win-win” cooperation scenarios (Leger et al., 2000). This behavior suggests that cooperative defect is an early manifestation of AD, but the pathologic mechanism remains elusive. Exploring this issue could assist in the early prevention and treatment of AD.

Rodents are social animals with excellent social intelligence and mutual assistance skills (Kingsbury et al., 2019; Leblanc and Ramirez, 2020). Recently, our group developed a cooperative drinking system that can quantitatively analyze cooperation willingness and efficiency among mice (Feng et al., 2021). In this study, by using this behavioral paradigm, we characterized age-dependent deficits in the cooperative ability of APP/PS1 transgenic AD mice.

The medial prefrontal cortex (mPFC) is a key regulatory region of social behaviors (Huang et al., 2020; Xing et al., 2021). Previous animal studies, including from our group, have demonstrated that social experience regulates mPFC myelination, and social interaction defects are related to mPFC hypomyelination (Cao et al., 2017; Liu et al., 2012; Makinodan et al., 2012). Based on this knowledge, we investigated whether cooperative declines of APP/PS1 mice were associated with impairments of mPFC myelination.

Miconazole is a broad-spectrum antifungal agent that inhibits cytochrome P450 fungal enzyme C-14α-demethylase (Hargrove et al., 2017). Recent literature has reported that miconazole stimulates differentiation of oligodendrocyte progenitor cells (OPCs) and repairs damaged myelin in mouse models of multiple sclerosis (MS) (Najm et al., 2015; Serrano-Pozo et al., 2011). We investigated whether treatment with miconazole could promote mPFC myelination and cooperative ability among APP/PS1 mice.

## METHODS

### Animals and Housing

In this experiment, we used 2-, 5- and 8-month-old male APP/PS1 (APPswePS1-dE9) transgenic mice and their wild-type (WT) littermates. Mice were maintained at a constant room temperature (18-22 °C), with controlled illumination (12:12-hour light/dark cycle), relative humidity of 30% to 50%, and food and water available ad libitum. All animal procedures were approved by the Institutional Animal Care and Use Committee (No. 1812054-3) of Nanjing Medical University.

### Miconazole Treatment

Two-month-old APP/PS1 mice and their WT littermates were randomly divided into 4 groups: WT control (WT-CON), WT-miconazole (WT-MIZ), APP/PS1-control (APP/PS1-CON), and APP-PS1-miconazole (APP/PS1-MIZ). Mice in the experimental group were injected intraperitoneally with 4 mg/mL miconazole (Sigma-Aldrich, #PHR1618) or vehicle (5% dimethyl sulfoxide [DMSO] in saline, 0.1 mL/10 g body weight) each day for 10 weeks (Najm et al., 2015). In the control group, 0.1 mL/10 g solvent only was injected intraperitoneally.

### Cooperative Drinking Test

Our laboratory designed the testing device of mouse cooperative behavior (Feng et al., 2021). Briefly, it consisted of 2 water taps controlled by a photoelectric switch located above the mouse cage (47 × 30 × 23 cm). The program included a 7-day training period and a 5-day testing period. Following 12 hours of water deprivation, mice were trained to drink water by touching 1 of the photoelectric switches, which turned on the tap. The mice were trained for 10 minutes per day. During the testing session, both photoelectric switches were turned on, and 2 mice previously housed in the same cage were put into the testing device. Both mice could drink water only when they contacted the water taps at the same time. Testing time was 5 minutes each day. Mouse activity was collected using a digital video camera connected to a computer-controlled system (Beijing Sunny Instruments Co Ltd, Beijing, China). The presence or absence of co-drinking water behavior, the time co-drinking water for the first time (drinking latency), and the number and duration of co-drinking episodes during the test were analyzed.

### Other Behavioral Tests

We performed social interaction tests, Y maze tests, and novel object recognition (NOR) tests according to our group’s previous studies (Cao et al., 2017; Zhang et al., 2020). The detailed procedures are available in the supplementary material.

### Primary OPC Culture and Differentiation

Cortices were isolated from the brain of postnatal 5- 7-day old C57Bl/6J mice and cut into small pieces after removing the meninges and blood vessels. The tissue was then incubated in Neurobasal with 20 to 30 U/mL papain and 2500 U deoxyribonuclease I at 37 °C for 20 minutes. The digested cells were filtered through a 40-µm mesh filter and resuspended in Neurobasal with 2% B27. The filtered cells were resuspended again in phosphate-buttered saline (PBS) containing Anti-O4 MicroBeads (Miltenyi Biotec, Beijing, China; #130-094-543) and incubated at 4 °C for 15 minutes. Beads were then captured by a column (Miltenyi Biotec, #130-042-201) to enrich the O4-positive primary OPCs (Flores-Obando et al., 2018). The OPCs were resuspended in the proliferation medium consisting of Dulbecco modified eagle medium/nutrient mixture-F12 (DMEM-F12) (Thermo Fisher, Waltham, MA, USA; #11320-033) with 1% N-2 (Thermo Fisher, #17502-048), 2% B27, 1% penicillin/streptomycin, and 40 ng/mL platelet-derived growth factor (R&D Systems, Shanghai, China; #1055-AA-050) at a density of 9000-15000 cells/cm_2_. The proliferation medium was replaced with fresh medium on the first day, and then half-changed every other day. After 8 to 9 days of proliferation, the proliferation medium was replaced by the differentiation medium, which consisted of DMEM-F12 with 1% N-2, 2% B27, 1% penicillin/streptomycin, 50 µg/mL insulin (Sigma-Aldrich, Beijing, China; #I-6634), 40 ng/mL triiodo tyrosine (Sigma-Aldrich, #T2877), and 1 ng/mL ciliary neurotrophic factor (R&D Systems, #557-NT) (Chen et al., 2007).

### Cultured OPCs and Oligodendrocytes Treated With Aggregating Aβ_1-42_/Miconazole

We plated OPCs onto a 12-well plate, while oligodendrocytes were plated onto 12-well, 24-well, or 6-well plates covered with poly-D-lysine hydrobromide (Sigma-Aldrich, #P6407) and laminin (Sigma-Aldrich, #114956-81-9) at a density of 9000 to 15 000 cells/cm^2^. The OPCs or oligodendrocytes treated by 2 μM aggregating human amyloid β peptide (Aβ_1-42_) (NJPeptide, Nanjing, China; #107761-42-2) for 24 hours, then either with or without treatment of 1 μM miconazole for 48 hours (Najm et al., 2015), followed by quantitative real-time polymerase chain reaction (qRT-PCR) (12-well plate), immunofluorescence (24-well plate), or Western blotting (6-well plate).

### Section Preparation

Mice were anesthetized with 80 mg/kg ketamine and 10 mg/kg xylazine, which was administered by intraperitoneal injection. They were then transcardially perfused with 0.9% saline by perfusion pump for 5 minutes, followed by 4% paraformaldehyde with or without 0.5% glutaraldehyde (for electron microscopy) for 15 minutes. Brains were removed, postfixed overnight at 4 °C, then dehydrated with a series of graded ethanol solutions and embedded in paraffin. Sagittal sections containing the mPFC were serially cut at 5 μm by a paraffin slicing machine for thioflavine-S staining or immunohistochemical staining. For electron microscopy, the forebrains were cut with a vibratome at 100 μm. The mPFC was trimmed and postfixed in 2% osmium, rinsed, dehydrated, and embedded in Epon 812. Ultrathin sections of 70 nm were cut, stained with uranyl acetate and lead citrate, and examined by a Jeol 1200EX electron microscope (Tokyo, Japan).

### Immunohistochemistry

Immunohistochemical staining was performed as previously described (Xu et al., 2015). Briefly, deparaffinized sections were incubated with 1 of the primary antibodies at 4 °C overnight (supplementary Table 1). Following PBS rinsing, sections were incubated with biotinylated-conjugated goat anti-mouse or rabbit immunoglobulin G (1:200, Vector Laboratories, Newark, CA, USA) for 1 hour at 37 °C, and then visualized with diaminobenzidine (Sigma-Aldrich). These sections were counterstained with Congo Red.

### Thioflavine-S Staining

Brain sections were incubated with 1% thioflavine-S (Sigma-Aldrich) for 5 minutes, washed by 70% ethanol for 5 minutes, and finally rinsed with distilled water. Brain sections were cover-slipped with antifluorescent quencher.

### Immunofluorescence

For immunofluorescence, frozen sections were blocked and permeabilized in blocking solution (2.5% bovine serum albumin in PBS) containing 0.1% Triton X for 1 hour at room temperature, incubated overnight at 4 °C with primary antibody antineuronal nuclei (NeuN) (1:500; Abcam, Cambridge, UK; #ab177487), anti-maltose binding protein (MBP) (1:400), glial fibrillary acidic protein (GFAP) (1:1000; Millipore, Burlington, MA, USA; #MAB360), ionized calcium binding adapter molecule 1 (Iba1) (1:1000; Wako, Osaka, Japan; #019-19741), or 6E10 (1:1000; BioLegend, San Diego, CA, USA; #SIG-39320). Next, the sections were incubated with the corresponding fluorescent probe-conjugated secondary antibodies (1:1000; Thermo Fisher, #A21202, #A21206, #A31572, #A31570, and #A31571) for 2 hours at room temperature while being protected from light. For primary oligodendrocyte staining, fixed coverslips were incubated with primary antibodies anti-O4 (1:200; R&D, #MAB1326) or anti-MBP (1:400). Nuclei were stained with 4,6-diamidino-2-phenylindole (Sigma, #D9542) at a 1:1000 dilution. Images were captured using an LSM700 confocal microscope (Zeiss, Berlin, Germany).

### Western Blotting

For Western blot analyses, the homogenized protein samples of the mPFC were loaded onto 10% to 16% Tris/tricine SDS gels and transferred to PVDF membranes (Kim, 2017). After blocking for 1 hour in 5% nonfat milk/TBST, the membranes were incubated at 4 °C overnight with 1 of the primary antibodies listed in supplementary Table 1. Horseradish peroxidase-conjugated secondary antibodies (Vector Laboratories) were used, and bands were visualized using the ECL plus detection system. Glyceraldehyde-3-phosphate dehydrogenase (GAPDH) was used as an internal reference for protein loading and transfer efficiency.

### Quantitative Real-Time PCR

Total RNA from the oligodendrocytes was extracted with Trizol according to the manufacturer’s instructions. We synthesized circulating DNA (cDNA) from 1 µg total RNA using the Maxima First Strand Synthesis Kit for RT-qPCR (Takara, Shiga, Japan; #RR047B). We performed qRT-PCR by amplifying cDNA for 40 cycles using the SYBR Green PCR master mix. Relative expression of messenger RNA for the target genes was calculated by the comparative 2^-ΔΔCT^ method using GAPDH as a control reference gene (Schmittgen and Livak, 2008). Primers for oligodendrocyte lineage transcription factor 2 (Olig2), Sox10, platelet-derived growth factor receptor α (PDGFRα), MBP, adenomatous polyposis coli clone (CC1) and GAPDH were synthesized by TsingKe (Beijing, China). All primer information is listed in (supplementary Table 2). Three values (for RNA extraction) in duplicate experiments were averaged to provide a mean value for each group.

### Image Analysis and Cell Counting

All brain slices were captured by a digital microscope (Leica Microsystems, Wetzlar, Germany). The same exposure time, saturation, and gain were used for each index. We used US National Institutes of Health ImageJ software to analyze the positive area of MBP, GFAP, Iba1, 6E10, and Thioflavine-S in the corresponding images. The number of NeuN-positive neurons in the mPFC was counted and presented as per mm^2^. The mean integrated optical density of MBP immunoreactive products in the mPFC, parietal association cortex (PtA), and hippocampus was also analyzed on immunohistochemical staining sections. Five sections per mouse and 6 mice per group were averaged to provide a mean value for each group. The electron micrographs were used to determine the extent of heterochromatin of individual oligodendrocyte lineage cells. The total nuclear area of each cell was calculated using the ImageJ software, and heterochromatin was selected using the threshold tool and reported as a percentage of total nuclear area (Castejón, 1984; García-Cabezas et al., 2016). The *g*-ratio of myelinated axons was also determined and calculated as the diameter of the axon divided by the diameter of the entire myelinated fiber, as previously described (Liu et al., 2012). A minimum of 10 myelinated axons per area of interest per animal were analyzed. All quantification was done blind to animal genotype and treatment. Using ImageJ, pictures were converted to 8-bit files with a set threshold. The center of a cell was defined manually, and Sholl analysis was performed by starting with a circle with a diameter of 0.05 μm and expanding the circles by 0.05 μm each time. Intersections per circle were counted using the ImageJ Sholl analysis. Results from each cell per conditions were integrated and analyzed using Microsoft Excel (Redmond, WA, USA) (Flygt et al., 2018).

### Statistical Analysis

All statistical data were presented as means (standard error of the mean [SEM]) and analyzed using SPSS, version 22.0, software and plotted by GRAPD PRISM, version 6.0. One-way analysis of variance (ANOVA) or 2-way ANOVA was performed for comparisons between multigroup studies, and Tukey post hoc tests were conducted only if the *F* in ANOVA achieved the necessary statistical significance level (*P* < .05). In addition, we performed Wilcoxon matched-pairs signed rank tests to compare the percentage of mice in each group that had successfully obtained the water via the photoelectric switch during the cooperative behavior tests. There was no significant variance in homogeneity. No data values were excluded in any statistical analysis.

## RESULTS

### Age-Dependent Decline in Cooperative Capability of APP/PS1 Mice

**Figure 1.**
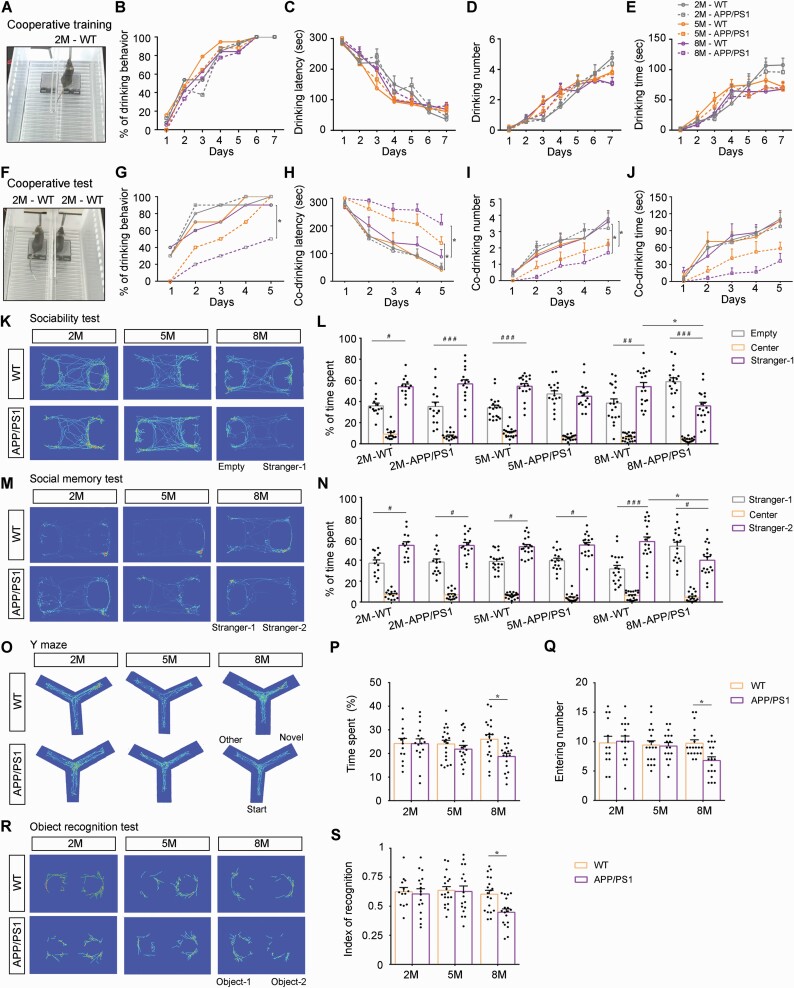
Cooperative behavior tests of APP/PS1 mice and wild-type (WT) mice at 2 months (2M), 5 months (5M), and 8 months (8M) of age. (A) A typical photograph of a 2-month-old mouse drinking water by touching 1 of the photoelectric switches during the training phase. (B) The percentage of mice in each group that learned to drink water during the 7-day training phase. Statistical graph showing (C) drinking latency, (D) drinking number, and (E) drinking time during the training period. (F) A photograph of a pair of 2-month-old WT mice drinking water together by touching the photoelectric switch at the same time during the testing phase. (G) The percentage of mice in each group that learned to co-drink water during the 5-day training period. Statistical graph showing (H) co-drinking latency, (I) co-drinking number, and (J) co-drinking time during the testing phase. Trajectory heat maps of the (K) social preference test and (M) social memory test. (L) The statistical graph showing the ratio of the percentage of time spent in the stranger-1 chamber vs the empty chamber in the social preference test. (N) Statistical graph showing the ratio of the percentage of time spent in the stranger-2 chamber vs the stranger-1 chamber during the social memory test. (O) Y maze cross-trajectory heat maps. (P) The percentage of time in the novel arm and (Q) entry numbers into the novel arm during the Y maze test. (R) Novel object recognition (NOR) cross-trajectory heat maps. (S) Identification of the NOR test index. Data in (B) and (G) are represented as a percentage and analyzed using the Wilcoxon matched-pairs signed rank test; other data are represented as mean (standard error of the mean) and analyzed by repeated-measures analysis of variance (ANOVA) with post hoc Student-Newman-Keuls test (C-E and H-J) or 2-way ANOVA followed by the Tukey post hoc test (L, N, P, Q, and S). 2M-WT: n = 13; 2M-APP/PS1: n = 16; 5M-WT: n = 19; 5M-APP/PS1: n = 17; 8M-WT: n = 19; 8M-APP/PS1: n = 18 in (B-E, L, N, P, Q, and S). n = 10 in each group that a pair of combinations is used and mice in the same litter are combined with each other (G-J). *P < .05, comparison between genotypes; ^#^*P* < .05; ^##^*P* < .01; ^###^*P* < .001, comparison between empty vs stranger-1 or empty vs stranger-2.

APP/PS1 mice aged 2 months, 5 months, and 8 months were used to observe and compare their cooperative performances in the co-drinking water task. Each mouse was given 5-minute daily water training for 7 days under the control of a photoelectric switch (Figure 1A), during which the presence or absence of drinking water behavior as well as the drinking latency, number, and times per day were counted. On the first day of training, neither 5-month-old nor 8-month-old APP/PS1 mice drank water successfully, while 8% to 18% of mice in the other groups used the photoelectric switch to drink water. As the training days increased, however, the percentage of mice able to drink water gradually increased in each group, all reaching 100% by day 6 (Figure 1B). Consistently, drinking latency decreased, and both drinking number and drinking time decreased with training days (Figure 1C-1E). On the seventh day, the above indexes were comparable between APP/PS1 mice and WT mice in each age group (supplementary Figure 1A-1C). Next, mice were randomly paired within groups to examine their cooperative ability for 5 days, 10 minutes/day (Figure 1F). On the first day, neither 5-month-old nor 8-month-old APP/PS1 mice had cooperative water drinking behavior, but at least 1/3 of the mouse pairs in the other groups acquired water successfully (Figure 1G). The percentage of cooperative drinking improved with increased training days, but the magnitude of 8-month-old APP/PS1 mice was lower than that of their age-matched WT littermates (*P* < .05). Specifically, the cooperative water drinking percentage of 8-month-old APP/PS1 mice on the fifth day was only 50%, while that of the other groups was more than 90% (Figure 1G). APP/PS1 mice and WT mice at 2 months of age were similar in drinking latency, drinking time, and drinking frequency during the 5-day test period, but 5-month-old APP/PS1 mice exhibited significantly longer co-drinking latency, shorter co-drinking time, and fewer co-drinking numbers than age-matched WT mice (*P* < .01, *P* < .05, *P* < .05, respectively). Up to the age of 8 months, the changes in the above indexes were more obvious between the 2 genotypes (*P* < .05, *P* < .05, *P* < .01, respectively) (Figure 1H-1J, supplementary Figure 1D-1F). Together, the above results revealed age-dependent cooperative defects in APP/PS1 mice.

Previous studies have found that APP/PS1 mice exhibit social interaction weaknesses in the 3-chamber test (Kosel et al., 2020; Pietropaolo et al., 2012), but the timing of this behavior abnormality has not been well investigated. APP/PS1 mice also exhibited progressive impairments in sociability, as revealed by an age-dependent decline in the preference for contact with an unfamiliar mouse (stranger-1), which was manifested in the decrease in stranger-1/empty ratio, and had significant differences with WT controls at the age of 8 months (*P* < .05) (Figure 1K and 1L). Similarly, during the second phase of the social memory test, we found that the APP/PS1 mice and WT mice at 2 and 5 months spent more time in the stranger-2 chamber than in the stranger-1 chamber, which manifested in the decrease in stranger-2/stranger-1 ratio, and there was no difference between the 2 genotypes (*P *> .05). The stranger-2/stranger-1 ratio decreased significantly in 8-month-old APP/PS1 mice, however, suggesting impaired social memory (*P* < .05) (Figure 1M and 1N).

We also compared cognitive function between APP/PS1 mice and WT mice at different ages using the Y maze test and NOR test (Figure 1O and 1R). Entrance number and time spent in the novel arm of the Y maze (Figure 1P and 1Q) and the cognitive index of the recognition of new objects (Figure 1S) were comparable between APP/PS1 mice and WT mice at 2 and 5 months of age. The above indexes significantly decreased in 8-month-old APP/PS1 mice compared with age-matched WT littermates (all *P* < .05), however, suggesting that cognitive function was impaired. Collectively, the above results revealed that cooperative dysfunction occurred before impairments in social interaction and spatial cognition during AD-like progress of APP/PS1 mice.

### Age-Dependent Hypomyelination in the mPFC of APP/PS1 Mice

**Figure 2.**
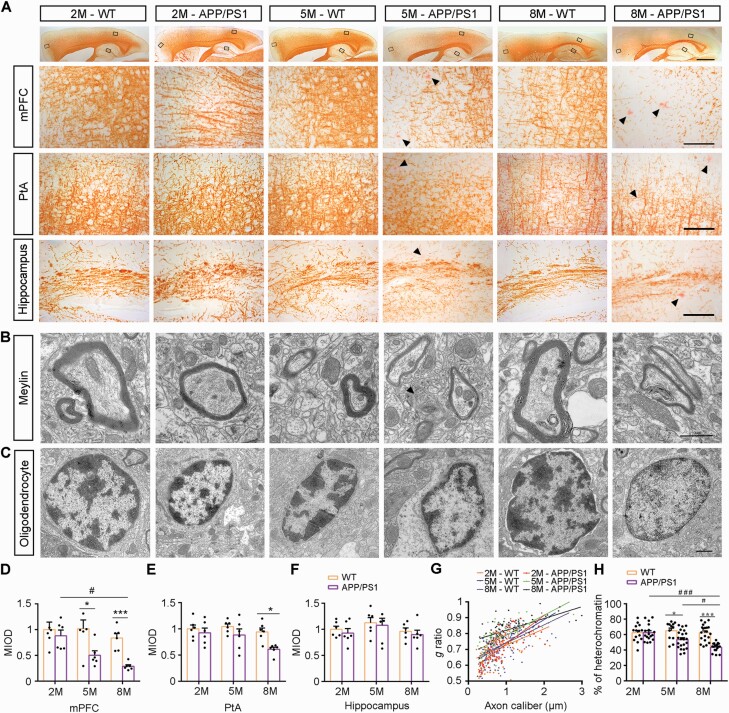
Impairment of myelination in the medial prefrontal cortex (mPFC) of APP/PS1 mice increased with age. (A) Immunohistochemical images of maltose binding protein (MBP)–positive myelin fibers in the forebrain of 2- (2M), 5- (5M), and 8-month-old (8M) APP/PS1 mice and wild-type (WT) mice, with high-power micrographs of the mPFC, the molecular layer of the hippocampus, and the parietal association cortex (PtA). Arrowheads show Congo Red–positive plaques. Scale bar: 1 mm (upper panel), 100 μm (lower panels). Representative electron microscopy images of (B) myelin sheath and (C) oligodendrocyte nuclear heterochromatin in the mPFC of 2M, 5M, and 8M APP/PS1 mice and WT mice. Scale bar: 1 μm. (D-F) The statistical diagram showing the mean integrated optical density (MIOD) of MBP expression in the (D) mPFC, (E) PtA, and (F) hippocampus of mice in each group (n = 6). (G) Scatter plot of *g* ratio values in 2M-WT (92 axons), 2M-APP/PS1 (68 axons), 5M-WT (89 axons), 5M-APP/PS1 (97 axons), 8M-WT (102 axons), and 8M-APP/PS1 (77 axons) mice (n = 6, at least 10 myelinated axons in each mouse mPFC area). (H) Dotted histogram showing the percentage of oligodendrocyte nuclear heterochromatin in the mPFC in 2M-WT (14 nuclei), 2M-APP/PS1 (17 nuclei), 5M-WT (12 nuclei), 5M-APP/PS1 (22 nuclei), 8M-WT (20 nuclei), and 8M-APP/PS1 (17 nuclei) mice (n = 6, at least 2 oligodendrocyte nuclei in each mouse mPFC area). All data are represented as mean (standard error of the mean). Data were analyzed using 2-way analysis of variance followed by the Tukey post hoc test. *P < .05 and ***P < .001, comparison between genotypes; ^#^*P* < .05 and ^###^*P* < .001, comparison among different ages.

Both in vivo and in vitro studies suggest that oligodendrocytes are more susceptible to the toxic effects of Aβ than neurons (Angeli et al., 2020; Butt et al., 2019; Roth et al., 2005). The previous literature reported that the mPFC regulates social behavior based on the integrity of myelin (Liu et al., 2012; Najm et al., 2015). Therefore, we investigated changes in myelin fibers in the mPFC as well as PtA and hippocampus of APP/PS1 mice at different ages through immunohistochemical staining for MBP and semiquantitative analysis. There was no significant difference in MBP immunoreactive products in the above 3 brain regions of APP/PS1 mice at 2 months of age, but MBP expression decreased in the mPFC at the age of 5 months and in the mPFC and PtA at 8 months of age compared with that in age-matched WT littermates (Figure 2A and 2D-2F). Western blot analysis confirmed decreases in MBP expression in the mPFC of 5- and 8-month-old APP/PS1 mice relative to age-matched WT mice (all *P* < .05) (supplementary Figure 2A and 2B). Therefore, ultrastructural changes of myelin sheaths and oligodendrocytes in the mPFC of APP/PS1 mice at different ages were examined by transmission electron microscopy. APP/PS1 mice at 2 months of age exhibited normal morphologic features of myelin sheaths and oligodendrocytes. There was no significant difference in the *g*-ratio for myelinated axons and the area percentage of heterochromatin in the oligodendrocyte nucleus between the 2 genotypes. At the age of 5 months, however, the thickness of myelin sheaths of APP/PS1 mice was significantly less than that of WT mice (*P *< .05), and oligodendrocyte heterochromatin was also significantly decreased (*P *< .05), suggesting that myelination maintenance was impaired. Notably, up to 8 months of age, hypomyelination was more evident in the mPFC of APP/PS1 mice (*P < *.001 for the *g-*ratio and percentage area of heterochromatin) (Figure 2B, 2C, 2G, and 2H).

**Figure 3.**
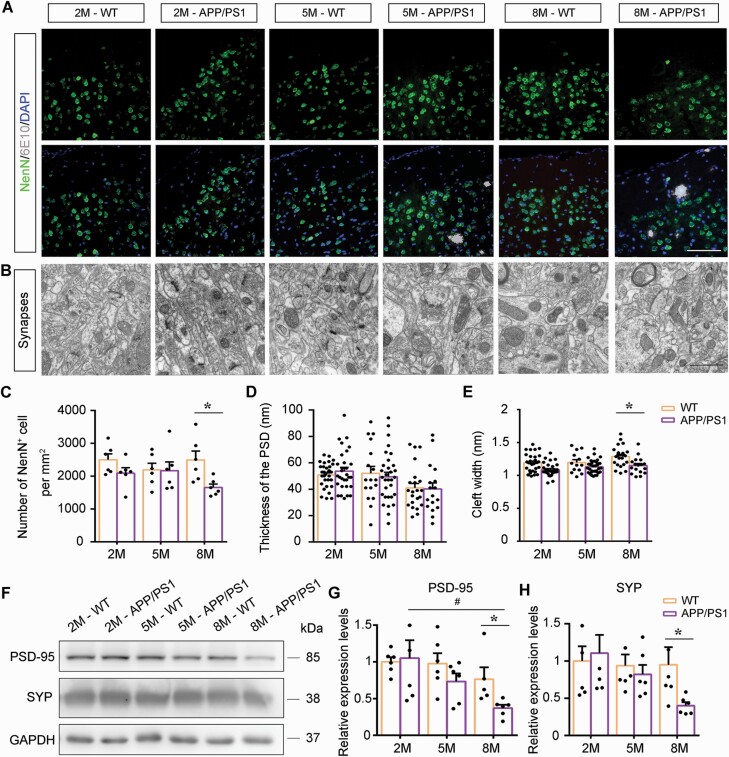
Neuron and synapse density in the medial prefrontal cortex (mPFC) of APP/PS1 mice and wild-type (WT) mice at 2 months (2M), 5 months (5M), and 8 months (8M) of age. (A) Antineuronal nuclei (NeuN) and 6E10 double-immunofluorescence show that there was neuron loss around the plaques (white areas) in APP/PS1 mice, but the overall density of neurons did not decrease significantly compared with age-matched WT mice. Scale bar: 100 μm. (B) The representative images of the mPFC synapses of APP/PS1 mice and WT mice at different ages. Scale bar: 1 μm. (C) Comparison of the number of neurons per unit area in each group (n = 6). (D, E) Graphs showing quantified analysis of postsynaptic density and synaptic cleft width in 2M-WT (35 synapses), 2M-APP/PS1 (39 synapses), 5M-WT (19 synapses), 5M-APP/PS1 (44 synapses), 8M-WT (25 synapses), and 8M-APP/PS1 (27 synapses) mice (n = 6, at least 3 synapses in each mouse mPFC area). (F) Representative Western blot bands and integral optical density analysis of (G) PSD-95 and (H) SYP expression levels in the mPFC of mice in each group (n = 6). All data are represented as mean (standard error of the mean). Data in (C-E) and (G, H) were analyzed using 2-way analysis of variance followed by the Tukey post hoc test. *P < .05, comparison between genotypes; ^#^*P* < .05, comparison among different ages. Abbreviations: DAPI, 4,6-diamidino-2-phenylindole; GAPDH, glyceraldehyde-3-phosphate dehydrogenase; PSD, postsynaptic density protein; SYP, synaptophysin.

In addition, the number of NeuN-positive neurons was comparable between APP/PS1 mice and WT controls at 2 and 5 months of age (Figure 3A and 3C). Electron microscopic images of synapses showed that postsynaptic density and synaptic cleft width were not different between the 2 genotype mice in the above age groups (Figure 3B, 3D, and 3E). The expression levels of postsynaptic density protein-95 (PSD-95) and synaptophysin (SYP) in the mPFC were also no different in the 2 groups (Figure 3F-3H), but degeneration of synapses plus loss of mPFC neurons and synaptic proteins were observed in 8-month-old APP/PS1 mice (all *P < *.05). Together, these results indicate that the early occurrence of cooperative malfunction of APP/PS1 mice could primarily be related to hypomyelination but not neuronal and synapse loss.

**Figure 4.**
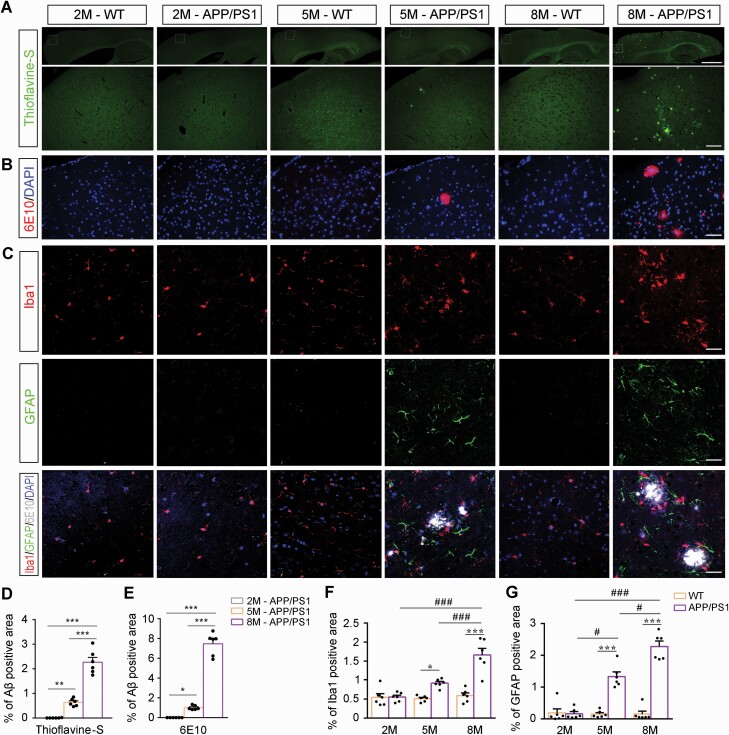
Aβ plaque deposition accompanied by reactive gliosis in the medial prefrontal cortex (mPFC) of APP/PS1 mice gradually increased with age. (A) Thioflavine-S staining and (B) immunofluorescence for 6E10 showing Aβ plaque deposition in the mPFC. Scale bar: 1 mm (upper panel) and 100 µm (lower panel). (C) Representative immunofluorescence images showing the expression of glial fibrillary acidic protein (GFAP)–positive astrocytes and ionized calcium binding adapter molecule 1 (Iba1)–positive microglia in the mPFC of mice in each group. Scale bar: 50 μm. Statistical graph showing deposition of (D) Thioflavine-S–positive and (E) 6E10-positive Aβ plaques in APP/PS1 mice and wild-type (WT) mice aged 2 months (2M), 5 months (5M), and 8 months (8M) of age. The statistical diagram showing the percentage of area occupied by (F) Iba1 (F) or (G) GFAP immunofluorescent signal in the mPFC of mice in each group. All data are represented as mean (standard error of the mean). n = 6 in each group. Data in (D) and (E) were analyzed using 1-way analysis of variance (ANOVA) followed by the Tukey post hoc test. Data in (F) and (G) were analyzed using 2-way ANOVA followed by the Tukey post hoc test. *P < .05, **P < .01, and ***P < .001, comparison between genotypes; ^#^*P* < .05 and ^###^*P* < .001, comparison among different ages. Abbreviation: DAPI, 4,6-diamidino-2-phenylindole.

We also determined whether impaired myelination in the mPFC of APP/PS1 mice was associated with Aβ plaque accumulation. Plaque deposition was observed in the mPFC of APP/PS1 mice from 5 months of age and was further evident at 8 months of age, as revealed by Thioflavine-S staining (Figure 4A and 4D) and 6E10 immunofluorescence stating (Figure 4B and 4E). Consistently, there were age-dependent increases in reactive gliosis in the mPFC of APP/PS1 mice (Figure 4C, 4F, and 4G). Together, the above data suggest that Aβ accumulation–related hypomyelination in the mPFC might be 1 pathologic mechanism for cooperative defects in APP/PS1 mice.

### Miconazole Treatment Improved Cooperative Ability in APP/PS1 Mice

Miconazole has proven effective in promoting precocious myelination in postnatal mouse brains as well as organotypic cerebellar slice cultures (Najm et al., 2015). Miconazole also enhances differentiation of cultured human OPCs into mature oligodendrocytes (Franklin, 2015). Therefore, we further determined whether abnormalities of social behaviors in APP/PS1 mice are the result of impairments in myelin maintenance and whether the deficiencies could be repaired through treatment with miconazole. Two-month-old APP/PS1 mice were given intraperitoneal miconazole for 10 weeks, then underwent cooperative ability, social interaction, and cognitive function assessments.

**Figure 5.**
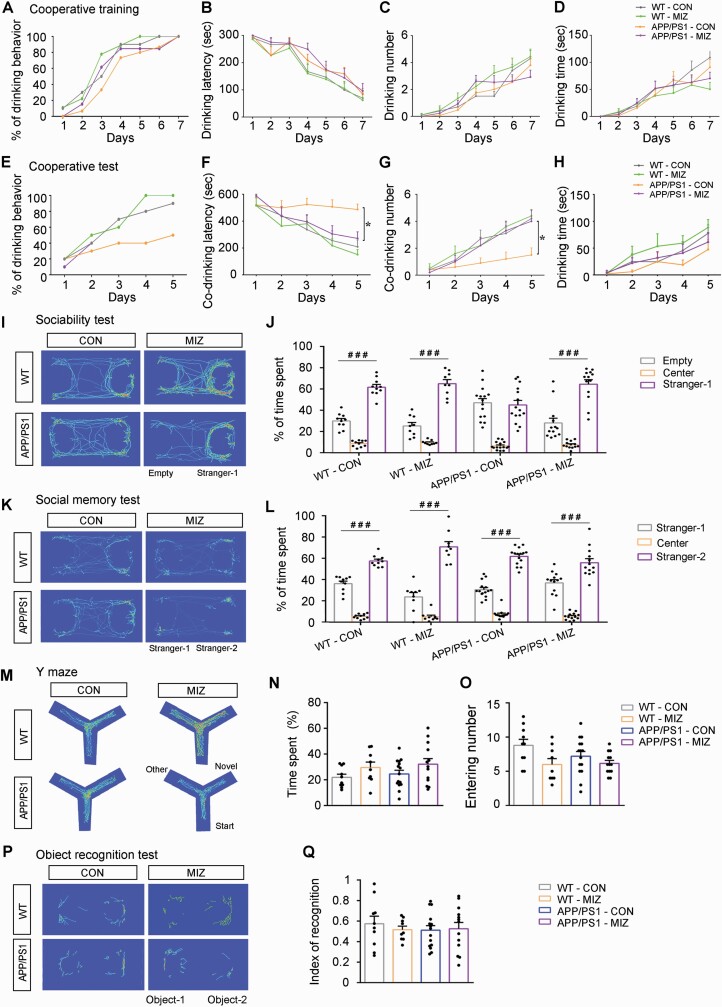
Miconazole therapy improved the cooperative ability of APP/PS1 mice. (A) The percentage of mice in each group that learned to drink water during the 7-day training phase. Statistical graph showing (B) drinking latency, (C) drinking number, and (D) drinking time during the cooperative training period. (E) The percentage of mice in each group that learned to co-drink water during the 5-day testing period. Statistical graph showing (F) co-drinking latency, (G) co-drinking number, and (H) co-drinking time during the testing phase. Trajectory heat maps of (I) the social preference test and (K) the social memory test. (J) Statistical graph showing the ratio of the percentage of time spent in the stranger-1 chamber vs the empty chamber during the social preference test. (L) Statistical graph showing the ratio of the percentage of time spent in the stranger-2 chamber vs the stranger-1 chamber during the social memory test. (M) Y maze cross-trajectory heat maps. (N) The percentage of time in the novel arm and (O) the entry numbers into the novel arm during the Y maze test. (P) Novel object recognition (NOR) cross-trajectory heat maps. (Q) Identification of the NOR test index. Data in (A) and (E) are represented as percentages and were analyzed using the Wilcoxon matched-pairs signed rank test; other data are represented as mean (standard error of the mean) and were analyzed using repeated-measures analysis of variance (ANOVA) with post hoc Student-Newman-Keuls test (B-D and F-H) or 2-way ANOVA followed by the Tukey post hoc test (J, L, N, O, and Q). Wild-type (WT)-control (CON): n = 10; WT-miconazole (MIZ): n = 9; APP/PS1-CON: n = 15; APP/PS1-MIZ: n = 13 in (A-D, J, L, N, O, and Q). n = 10 in each group in which a pair of combinations were used; mice from the same litter were combined with each other in (E-H). *P < .05 and **P < .01, comparison between miconazole (MIZ) treatment and dimethyl sulfoxide control. ^#^*P* < .05, ^##^*P* < .01, and ^###^*P* < .001, comparison between empty vs stranger-1 or empty vs stranger-2.

Four groups of mice with or without miconazole treatment were trained to use the photoelectric switch–controlled drinking device. On the first day of training, APP/PS1 mice with or without miconazole therapy did not obtain water reward successfully, while 10% of mice in the other groups successfully used the photoelectric switch to drink water. As the number of training days increased, however, the percentage of mice able to drink water gradually increased in each group, all reaching 100% by day 7 (Figure 5A). Drinking latency also gradually decreased, while the time and frequency of drinking water increased gradually as training continued; results were similar between APP/PS1 mice with and without miconazole therapy (*P > *.05, *P > *.05, *P> *.05, respectively) (Figure 5B-5D; supplementary Figure 3A-3C). The cooperative ability test showed that the percentage of cooperative drinking improved in APP/PS1 mice as the number of testing days increased, and the difference was not significant different between APP/PS1 mice on and not on miconazole (Figure 5E). The cooperative water drinking percentage of APP/PS1 mice was only 50% on the fifth day, however, while in APP/PS1 mice receiving miconazole treatment it was about 90% (Figure 5E). Further quantitative analysis showed that co-drinking latency among APP/PS1 mice in the miconazole treatment group was significantly lower than that of the control group (*P* *<* .01) (Figure 5F; supplementary Figure 3D). The co-drinking number also significantly increased in the APP/PS1-MIZ group (*P* *<* .05) (Figure 5G; supplementary 3E), although increases in co-drinking time were not significant (Figure 5H; supplementary Figure 3F) compared with the APP/PS1-CON group. These results demonstrated that miconazole partially improves cooperative ability among APP/PS1 mice.

The 3-chamber test revealed that saline-treated APP/PS1 mice did not exhibit a preference for stranger-1, but miconazole-treated APP/PS1 mice spent more time contacting stranger-1 than staying in the empty chamber (*P* *<* .01), indicating that social preference behavior partially improved (Figure 5I and 5J). The mice in each group showed a significantly longer exposure time to stranger-2 than to stranger-1, and miconazole therapy failed to further enhance social memory capability (Figure 5K and 5L). In addition, behavioral performances in the Y maze test and recognition of new objects were comparable between miconazole-treated APP/PS1 mice and APP/PS1 controls (Figure 5M-5Q).

### Miconazole Treatment Ameliorated Myelin Damage Without Affecting Aβ Load in APP/PS1 Mice

**Figure 6.**
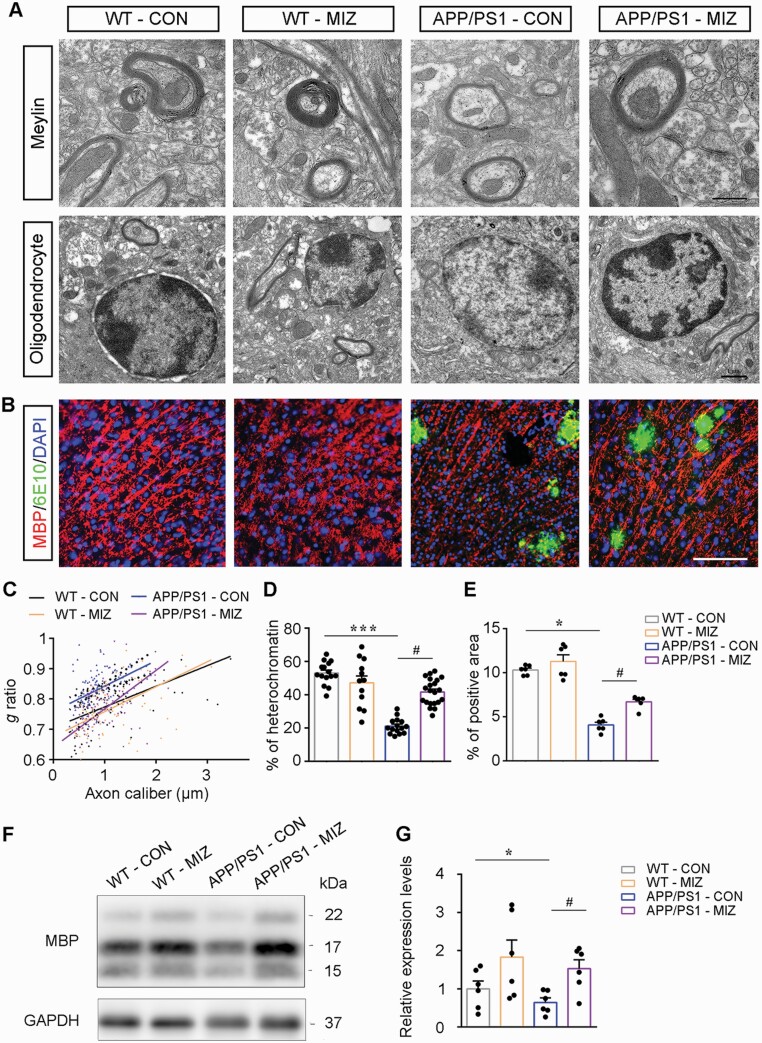
Miconazole (MIZ) therapy ameliorated myelin damage in 5-month-old APP/PS1 mice. (A) Representative electron microscopy images showing myelin sheath and oligodendrocyte nuclear heterochromatin in the medial prefrontal cortex (mPFC) of APP/PS1 mice and wild-type (WT) mice with or without miconazole treatment. Scale bar: 500 nm (top), 1 μm (bottom). (B) Double-immunofluorescence of maltose binding protein (MBP) and 6E10 show that miconazole treatment increased MBP-positive myelin fibers in the mPFC of APP/PS1 mice. Scale bar: 50 μm. (C) Scatter plot of the myelin sheath *g* ratio in WT-control (CON) (95 axons), WT-MIZ (105 axons), APP/PS1-CON (115 axons), and APP/PS1-MIZ (121 axons) mice (n = 6, at least 20 myelinated axons in each mouse mPFC area). (D) The percentage of oligodendrocyte nuclear heterochromatin in the mPFC in WT-CON (15 nuclei), WT-MIZ (12 nuclei), APP/PS1-CON (15 nuclei), and APP/PS1-MIZ (21 nuclei) mice (n = 6, at least 2 oligodendrocyte nuclei in each mouse mPFC area). (E) Statistical diagram showing the percentage of area occupied by MBP immunofluorescent signal in the mPFC of mice in each group (n = 6). (F, G) Representative Western blot bands and the integral optical density analysis of MBP expression in the mPFC (n = 6). All data are represented as mean (standard error of the mean). Data in (D, E) and (G) were analyzed using 2-way analysis of variance followed by the Tukey post hoc test. *P < .05 and ***P < .001, comparison between genotypes; ^#^*P*< .05 and ^###^*P* < .001, comparison between MIZ treatment and dimethyl sulfoxide control. Abbreviations: DAPI, 4,6-diamidino-2-phenylindole; GAPDH, glyceraldehyde-3-phosphate dehydrogenase.

We determined whether miconazole treatment, which improved cooperative capability of APP/PS1 mice, was associated with mPFC myelin repair. As expected, the *g*-ratio of myelinated axons was reduced, and the area percentage of oligodendrocyte heterochromatin increased in miconazole-treated APP/PS1 mice compared with saline-treated APP/PS1 mice (both *P* *<* .001) (Figure 6A, 6C, and D). Immunofluorescence, immunohistochemistry, and Western blot showed increases in MBP expression in the mPFC of APP/PS1 mice subsequent to miconazole treatment (all *P* *<* .05) (Figure 6B and 6E-6G; supplementary Figure 4A and 4B). Miconazole did not significantly affect MBP immunoreactivity in the PtA and hippocampus of APP/PS1 mice (supplementary Figure 4A, 4C, and 4D). In addition, the above indexes of myelination in WT mice treated with miconazole were not significantly changed, suggesting that miconazole did not affect baseline myelination. Miconazole treatment also did not affect the density and morphologic profile of synapses and expression levels of PSD-95 and SYP in the mPFC of APP/PS1 mice and WT mice (supplementary Figure 5A-5E).

**Figure 7.**
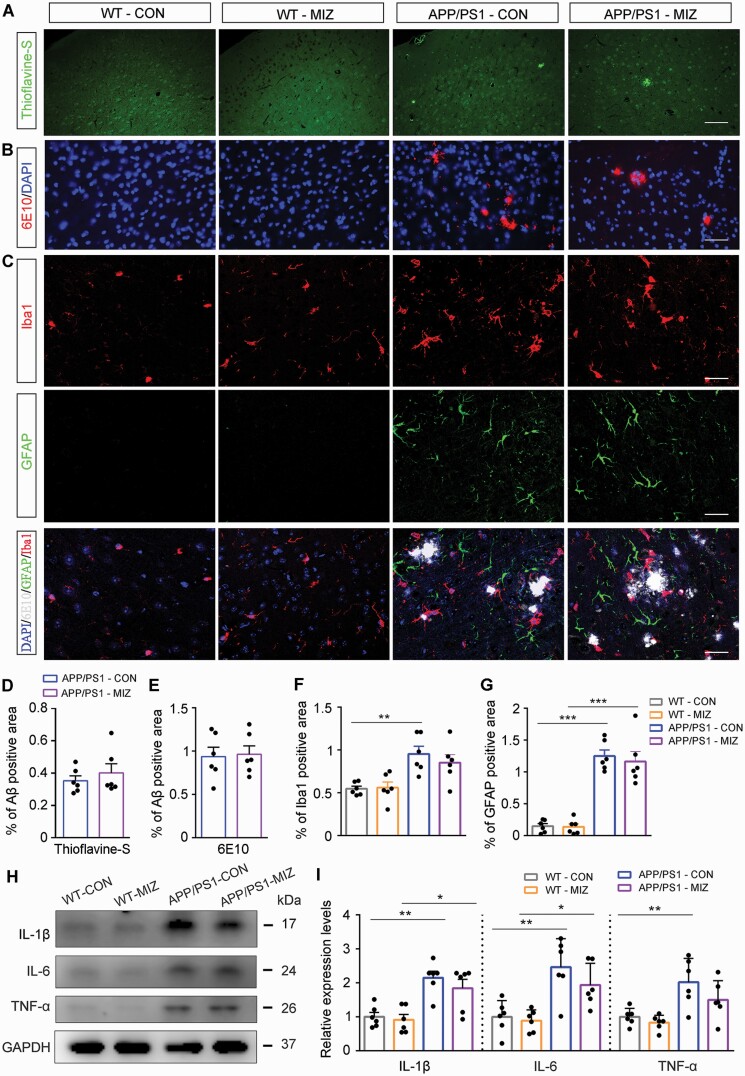
Miconazole (MIZ) failed to reduce the deposition of Aβ plaques and neuroinflammation in the medial prefrontal cortex (mPFC) of APP/PS1 mice. (A) Thioflavine-S staining and (B) immunofluorescence for 6E10 staining show Aβ plaque deposition in the mPFC of APP/PS1 mice with or without miconazole treatment. Scale bar: 100 µm. (C) Representative immunofluorescence images showing the expression of glial fibrillary acidic protein (GFAP)–positive astrocytes and ionized calcium binding adapter molecule 1 (Iba1)–positive microglia in the mPFC of mice in each group. Scale bar: 50 μm. Quantification of (D) Thioflavine-S–positive Aβ plaques and (E) 6E10-positive Aβ plaques in APP/PS1 mice with or without miconazole treatment. Statistical diagram showing the percentage of area occupied by (F) Iba1 or (G) GFAP immunofluorescent signal in the mPFC of mice in each group. (H) Representative Western blot bands and (I) the integral optical density analysis of interleukin (IL)-1β, IL-6, and tumor necrosis factor-α (TNF-α). All data are represented as mean (standard error of the mean). n = 6 in each group. Data were analyzed by 2-way analysis of variance followed by the Tukey post hoc test. *P < .05, **P < .01, and ***P < .001, comparison between genotypes. Abbreviations: CON, control; DAPI, 4,6-diamidino-2-phenylindole; GAPDH, glyceraldehyde-3-phosphate dehydrogenase; WT, wild type.

We further determined whether the myelin repair that miconazole promoted was also associated with reduced Aβ load. Thioflavine-S staining and 6E10 immunofluorescent results revealed that miconazole did not reduce Aβ plaque deposition in the mPFC of APP/PS1 mice (Figure 7A, 7B, 7D, and 7E). There was also no significant difference between miconazole treatment and DMSO control groups in expression levels of Aβ production-, transport-, and clearance-related markers, including ADAM10, BACE1, PS1, LRP1, and IDE (supplementary Figure 6A-F). In addition to repairing myelin, miconazole has been shown to inhibit neuroinflammation (Gong et al., 2022; Tsutsui et al., 2018; Yeo et al., 2020). Therefore, we determined whether miconazole has anti-inflammatory effects in APP/PS1 mice. The results showed that miconazole mildly reduced the activation of Iba1-positive microglia and GFAP-positive astrocytes in APP/PS1 mice, but the reduction did not reach a significant level (Figure 7C, 7F, and 7G). Consistently, there were no significant differences in the expression levels of inflammatory factors interleukin-1β (IL-1β), IL-6, and tumor necrosis factor-α in the mPFC homogenate between the 2 groups (Figure 7H and 7I). Taken together, these results suggest that miconazole enhances mPFC myelination, which may be the chief pharmacologic basis for improving cooperative ability among APP/PS1 mice.

### Miconazole Attenuated Toxic Effects of Aβ on Cultured Oligodendrocytes

**Figure 8.**
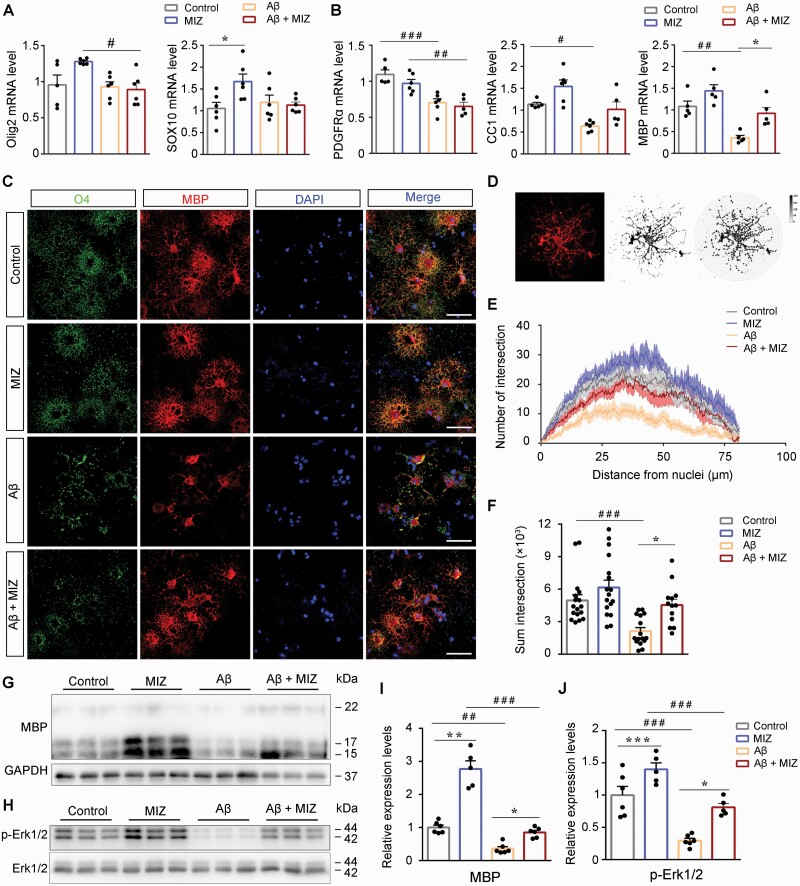
Miconazole (MIZ) improved maturation of cultured oligodendrocytes exposed to Aβ. Quantitative real-time polymerase chain reaction evaluating altered expression of differentiating markers of oligodendrocytes by incubated with 2 μM Aβ for 24 hours followed by 1 μM miconazole for an additional 48 hours in (A) oligodendrocyte progenitor cells and (B) primary oligodendrocytes. The expression level of these markers is normalized to glyceraldehyde-3-phosphate dehydrogenase (GAPDH) (n = 6 in A; n = 5 in B). (C) Primary oligodendrocytes were incubated with 2 μM aggregating human amyloid β peptide (Aβ_1-42_) for 24 hours followed by 1 μM miconazole for additional 48 hours. The expression of maltose binding protein (MBP) and oligodendrocyte marker (O4) was detected by immunofluorescence. Scale bar: 50 μm. (D) Statistical diagram of the number of oligodendrocyte branch intersections. (E, F) Sholl analysis identified that Aβ_1-42_ reduced intersections of concentric circles with the oligodendrocyte processes, which was corrected by MIZ treatment (17 cells for control, 17 cells for MIZ, 16 cells for Aβ_1-42_, and 13 cells for Aβ_1-42_ + MIZ) (n = 5, at least 2 different cells are randomly selected from each well). (G) Representative Western-blot bands and (I) integral optical density analysis of MBP expression in differentiating oligodendrocytes treated with Aβ_1-42_ or/and MIZ (n = 6). (H) Representative Western-blot bands and (J) integral optical density analysis of phosphor-extracellular-signal regulated kinase (p-Erk) expression in differentiating oligodendrocytes treated with Aβ_1-42_ or/and MIZ (n = 6). All data are represented as mean (standard error of the mean). Data in (A, B, F, I, and J) were analyzed by 2-way analysis of variance followed by the Tukey post hoc test. *P < .05, **P < .01, and ***P < .001, comparison between mice with and without MIZ treatment; ^#^*P* < .05, ^##^*P* < .01, and ^###^*P* < .001, comparison between mice with and without Aβ treatment. Abbreviations: CC1, adenomatous polyposis coli clone; DAPI, 4,6-diamidino-2-phenylindole; mRNA, messenger RNA; PDGFRɑ, platelet-derived growth factor receptor α; Olig2, oligodendrocyte lineage transcription factor 2.

To further confirm the protective effect of miconazole against Aβ-induced oligodendrocyte damage, mPFC mouse pup tissue was cultured in vitro to obtain OPCs, which was then induced to differentiation into mature oligodendrocytes. The OPCs or oligodendrocytes were treated by 2 μM aggregating Aβ_1-42_ for 24 hours, followed by either miconazole treatment or lack of miconazole for 48 hours. The expression levels of OPC markers in the proliferation and differentiation phases were then detected by qRT-PCR. The results showed that Aβ_1-42_ treatment had little effect on OPC proliferative-phase markers Olig2 and SOX10 (Butt et al., 2019) but reduced the expression of PDGFRα, CC1 and MBP in primary oligodendrocytes (*P* < .01, *P* < .05, *P* < .01, respectively). Combined treatment of Aβ_1-42_ and miconazole had no effect on the Olig2 and SOX10 gene expression levels in the OPCs (Figure 8A) but significantly upregulated MBP expression in oligodendrocytes (*P* < .01) (Figure 8B). In addition, miconazole treatment increased Olig2 and SOX10 expression only in OPCs, with no significant effects on the expression levels of PDGFRα, CC1, or MBP in oligodendrocytes. Immunofluorescence consistently demonstrated that Aβ_1-42_ dramatically reduced the process complexity, plus O4 and MBP expression, in oligodendrocytes, which were markedly reversed by miconazole treatment (*P* < .01) (Figure 8C). Miconazole-treated oligodendrocytes at the baseline condition did not affect oligodendrocyte morphology or O4 and MBP expression. Quantitative data revealed that the number and length of MBP-positive cell processes in the Aβ_1-42_–treated group were significantly lower than those in the control group (*P* < .01), while this damage was significantly ameliorated in the miconazole-treated group (*P* < .05) (Figure 8E and 8F). Western blot also demonstrated that miconazole partially rescued decreased MBP expression levels in cultured oligodendrocytes that were exposed to Aβ_1-42_ (*P* < .01) (Figure 8G and 8I). The above results indicate that miconazole could attenuate the toxic effects of Aβ on mature oligodendrocytes.

Previous studies suggest that Aβ inhibits mitogen-activated protein kinase (MAPK) signaling pathway–related survival pathways in culture neurons and oligodendrocytes (Florent et al., 2006; Ju et al., 2005), while miconazole can directly induce OPCs to differentiate into mature oligodendrocyte cells by inducing phosphorylation of ERK1/2 (Najm et al., 2015). We further determined whether the MAPK pathway is also involved in miconazole against the toxic effects of Aβ on mature oligodendrocytes. As expected, Aβ treatment of oligodendrocytes showed a decrease in ERK1/2 phosphorylation, and miconazole partially reversed this reduction (Figure 8H and 8J).

## DISCUSSION

As the world’s population continues to age, the lack of AD therapy has become a crucial impediment to many individuals, but little progress has been achieved (Ferrer, 2012). The primary reason for this situation is that the onset of AD is very subtle. When patients begin to develop symptoms, the brain has already undergone irreversible and deleterious neurodegenerative alterations (Leng and Edison, 2021), rendering current therapies ineffective (Henstridge et al., 2019). Therefore, it is urgent that we find early therapeutic targets to delay or even prevent disease onset.

APP/PS1 mice are the most commonly used AD model animals to simulate progressive spatial cognitive dysfunction as well as abnormal mental and social behaviors in patients with AD (Pietropaolo et al., 2012). Consistently, the present results revealed that 8-month-old APP/PS1 mice showed cognitive and social interaction deficits. To the best of our knowledge, however, cooperative alterations in APP/PS1 mice have not been previously reported. We examined the cooperative phenotype of APP/PS1 mice using a cooperative drinking behavior model that our group designed (Feng et al., 2021). Not only do the mice need to become familiar with the new way of drinking water, but they also need to interact and coordinate their actions to successfully complete the cooperative task. This behavioral testing strategy accurately reflects the inherent nature of cooperative behavior—that is, individuals may gain benefits that they cannot obtain by acting alone (Henry et al., 2016; Tomasello and Vaish, 2013). Five-month-old APP/PS1 mice displayed normal spatial, shape, and social memory, as revealed by the Y maze test, NOR test, and 3-chamber test. They also had no apparent learning defects in the process training to drink water with photoelectric switches, but their ability to get a water reward through the cooperative mode was significantly impaired. This means that in the process of implementing cooperative behavior, multiple brain functions, such as emotion, empathy, and communication, are involved to achieve mutual benefit. Our finding is in agreement with clinical reports revealing that before the onset of cognitive dysfunction, cooperation initiative and altruism of patients with AD are noticeably impaired (Leger et al., 2000; Mohs et al., 2000).

Cooperative behavior is essential to the survival of social animals (Clutton-Brock, 2021; Dale et al., 2020). Brain regions regulating cooperative behavior include the amygdala, striatum, nucleus accumbens, hippocampus, and mPFC (Cardinal et al., 2002; Omar et al., 2011). The mPFC receives input from these brain regions, such as serotonin and dopaminergic afferent fibers, and plays an essential integrating role in the establishment and maintenance of cooperative behavior (Bambico et al., 2015; Han et al., 2011).Therefore, we selected this brain region to explore the pathologic basis of APP/PS1 mice with cooperative behavior abnormality. Our results indicate that the pathologic basis of cooperative ability declines may be related to an impairment of long-term myelination maintenance in the mPFC caused by Aβ accumulation.

Myelin is a scallion-like structure formed by oligodendrocyte processes surrounding axons of central nervous system neurons, thereby making signal transduction more rapid and effective (Fields, 2014; Peles and Salzer, 2000; Young et al., 2013). Compared with neurons, oligodendrocytes are even more vulnerable to various stresses, including hypoxia (Ma et al., 2015), oxidative stress (Husain and Juurlink, 1995), and neuroinflammatory responses (Stephenson et al., 2018). This reality is supported by the fact that in normal aging and early stages of AD, white matter atrophy is more apparent than gray matter area damage, the location of the neuronal soma (Butt et al., 2019). Aβ is toxic to oligodendrocytes through a variety of mechanisms, such as oxidative stress (Nasrabady et al., 2018), mitochondrial DNA damage (Hsu et al., 2010), and apoptosis (Cai and Xiao, 2016; Song et al., 2019). A recent study reported that aggregating Aβ causes premature senescence of OPCs, subsequently hampering their differentiation into mature oligodendrocytes (Zhang et al., 2019). Therefore, myelin damage in APP/PS1 mice may be related to the accumulation of Aβ.

Moreover, a body of evidence shows that oligodendrocyte maturation and long-term maintenance of myelination are dependent on normal social experiences (Liu et al., 2012; Makinodan et al., 2012). Previous studies, including our own work, suggest that various social stresses, such as social isolation, can exacerbate the damage to myelin integrity in the mPFC of aged mice and APP/PS1 mice but can be rescued by an enriched physical environment (Cao et al., 2018; Crombie et al., 2021; Wang et al., 2018). The present data suggest that accumulation of Aβ in the mPFC during the early stages of APP/PS1 mouse life cycles impairs oligodendrocyte myelination, subsequently leading to social cooperative decline. Based on this finding, it is necessary to determine whether social interaction and cooperation training could repair myelin damage in APP/PS1 mice, consequently blocking this malicious pathophysiologic cycle between hypomyelination and social behavior defects.

Previous studies on demyelination have focused on immunosuppressants, such as glucocorticoids, to mitigate immune attacks during acute episodes (Lizak et al., 2017), but this strategy has not shown a significant effect on the remyelination process (Kumar et al., 1989). Therefore, recent studies have focused on developing drugs targeting myelin regeneration. Miconazole has been shown to repair damage to the myelin sheath in MS without an inhibitory effect on the immune system (Najm et al., 2015). We observed miconazole’s potential to produce a repairing effect on myelin sheath in the early AD-like pathology of APP/PS1 mice. The results indicated that the cooperative ability of APP/PS1 mice significantly improved following treatment with miconazole, which is associated with improved myelination in the mPFC. Our data also suggest that miconazole treatment does not ameliorate Aβ deposition in APP/PS1 mice. Miconazole has been reported to reduce neuroinflammation in experimental autoimmune encephalomyelitis (Tsutsui et al., 2018), an epilepsy model (Gong et al., 2022), and lipopolysaccharide-induced memory loss (Yeo et al., 2020). We did not, however, find decreased levels of inflammatory factors in the mPFC of APP/PS1 mice treated with miconazole. This finding suggests that miconazole treatment might be not sufficient to combat persistent reactive gliosis caused by chronic progressive accumulation of Aβ.

In vitro data confirmed that miconazole improves OPC differentiation and attenuates the toxic effects of Aβ on cultured oligodendrocytes. In agreement with this finding, previous studies suggested that miconazole can directly induce OPCs to differentiate into mature oligodendrocyte cells by inducing phosphorylation of ERK1/2 (Najm et al., 2015). We demonstrated that miconazole partially reverses inhibitions of MAPK transduction in cultured oligodendrocytes exposed to Aβ. Whether other mechanisms are involved in miconazole protecting against Aβ toxicity in cultured oligodendrocytes remains to be determined.

The current study has some limitations. Only male APP/PS1 mice were used to investigate the protective effects of miconazole on cooperative dysfunctions. Previous studies reported that female APP/PS1 mice develop Aβ plaque and have high peripheral Aβ levels at younger ages than male littermates (Ordóñez-Gutiérrez et al., 2015; Wang et al., 2003). Further research is warranted to determine whether sex differences affect the efficacy of miconazole in the treatment of AD-like pathologic and behavioral alterations. In addition to the mPFC, which brain regions are involved in the current cooperative drinking behavior needs to be clarified. In this study, we did not explore the protective effects of miconazole on social cooperation and myelination in the middle- and late-stage of AD-like pathology among APP/PS1 mice (ie, when overt myelin degeneration occurs).

## CONCLUSIONS

**Figure 9.**
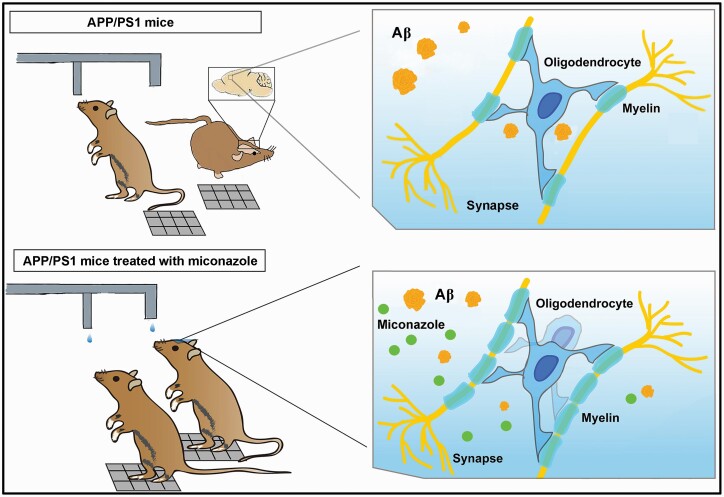
Schematic display of how miconazole promotes the cooperative ability of a mouse model of Alzheimer disease.

The present study demonstrated an impairment of cooperative ability in APP/PS1 mice associated with mPFC myelin degeneration. Miconazole treatment improves cooperative function and mPFC myelination of this AD mouse model (Figure 9). The potential for this new strategy as an early intervention for patients with AD remains to be validated in clinical trials.

## Supplementary Material

pyac061_suppl_Supplementary_Data

pyac061_suppl_Supplementary_Figure_S1

pyac061_suppl_Supplementary_Figure_S2

pyac061_suppl_Supplementary_Figure_S3

pyac061_suppl_Supplementary_Figure_S4

pyac061_suppl_Supplementary_Figure_S5

pyac061_suppl_Supplementary_Figure_S6

pyac061_suppl_Supplementary_Material

## References

[CIT0001] Angeli S , Kousiappa I, Stavrou M, Sargiannidou I, Georgiou E, Papacostas SS, Kleopa KA (2020) Altered expression of glial gap junction proteins Cx43, Cx30, and Cx47 in the 5XFAD model of Alzheimer’s disease. Front Neurosci 14:582934. 33117125 10.3389/fnins.2020.582934 PMC7575794

[CIT0002] Bambico FR , Lacoste B, Hattan PR, Gobbi G (2015) Father absence in the monogamous California mouse impairs social behavior and modifies dopamine and glutamate synapses in the medial prefrontal cortex. Cereb Cortex 25:1163–1175. 24304503 10.1093/cercor/bht310

[CIT0003] Butt AM , Chacon De La Rocha I, Rivera A (2019) Oligodendroglial cells in Alzheimer’s disease. Adv Exp Med Biol 1175:325–333. 31583593 10.1007/978-981-13-9913-8_12

[CIT0004] Cai Z , Xiao M (2016) Oligodendrocytes and Alzheimer’s disease. Int J Neurosci 126:97–104. 26000818 10.3109/00207454.2015.1025778

[CIT0005] Cao M , Pu T, Wang L, Marshall C, He H, Hu G, Xiao M (2017) Early enriched physical environment reverses impairments of the hippocampus, but not medial prefrontal cortex, of socially-isolated mice. Brain Behav Immun 64:232–243. 28412142 10.1016/j.bbi.2017.04.009

[CIT0006] Cao M , Hu PP, Zhang YL, Yan YX, Shields CB, Zhang YP, Hu G, Xiao M (2018) Enriched physical environment reverses spatial cognitive impairment of socially isolated APPswe/PS1dE9 transgenic mice before amyloidosis onset. CNS Neurosci Ther 24:202–211. 29274291 10.1111/cns.12790 PMC6490083

[CIT0007] Cardinal RN , Parkinson JA, Hall J, Everitt BJ (2002) Emotion and motivation: the role of the amygdala, ventral striatum, and prefrontal cortex. Neurosci Biobehav Rev 26:321–352. 12034134 10.1016/s0149-7634(02)00007-6

[CIT0008] Castejón OJ (1984) Low resolution scanning electron microscopy of cerebellar neurons and neuroglial cells of the granular layer. Scan Electron Microsc(pt 3):1391–1400. 6505621

[CIT0009] Chen Y , Balasubramaniyan V, Peng J, Hurlock EC, Tallquist M, Li J, Lu QR (2007) Isolation and culture of rat and mouse oligodendrocyte precursor cells. Nat Protoc 2:1044–1051. 17546009 10.1038/nprot.2007.149

[CIT0010] Clutton-Brock T (2021) Social evolution in mammals. Science 373:eabc9699. 34529471 10.1126/science.abc9699

[CIT0011] Crombie GK , Palliser HK, Shaw JC, Hodgson DM, Walker DW, Hirst JJ (2021) Effects of prenatal stress on behavioural and neurodevelopmental outcomes are altered by maternal separation in the neonatal period. Psychoneuroendocrinology 124:105060. 33333379 10.1016/j.psyneuen.2020.105060

[CIT0012] Dale R , Marshall-Pescini S, Range F (2020) What matters for cooperation? The importance of social relationship over cognition. Sci Rep 10:11778. 32678194 10.1038/s41598-020-68734-4 PMC7366628

[CIT0013] Feng W , Zhang Y, Wang Z, Wang T, Pang Y, Zou Y, Huang H, Sheng C, Xiao M (2021) A water-reward task assay for evaluating mouse mutualistic cooperative behavior. bioRxiv. Advance online publication (not peer reviewed). Retrieved 21 Sept 2022. doi: 10.1101/2021.02.06.430037.

[CIT0014] Ferrer I (2012) Defining Alzheimer as a common age-related neurodegenerative process not inevitably leading to dementia. Prog Neurobiol 97:38–51. 22459297 10.1016/j.pneurobio.2012.03.005

[CIT0015] Fields RD (2014) Myelin formation and remodeling. Cell 156:15–17. 24439366 10.1016/j.cell.2013.12.038 PMC5017146

[CIT0016] Flores-Obando RE , Freidin MM, Abrams CK (2018) Rapid and specific immunomagnetic isolation of mouse primary oligodendrocytes. J Vis Exp (135):57543. 10.3791/57543 PMC6101296 29863670

[CIT0017] Florent S , Malaplate-Armand C, Youssef I, Kriem B, Koziel V, Escanyé MC, Fifre A, Sponne I, Leininger-Muller B, Olivier JL, Pillot T, Oster T (2006) Docosahexaenoic acid prevents neuronal apoptosis induced by soluble amyloid-beta oligomers. J Neurochem 96:385–395. 16300635 10.1111/j.1471-4159.2005.03541.x

[CIT0018] Flygt J , Ruscher K, Norberg A, Mir A, Gram H, Clausen F, Marklund N (2018) Neutralization of interleukin-1β following diffuse traumatic brain injury in the mouse attenuates the loss of mature oligodendrocytes. J Neurotrauma 35:2837–2849. 29690837 10.1089/neu.2018.5660 PMC6247990

[CIT0019] Franklin RJ (2015) Regenerative medicines for remyelination: from aspiration to reality. Cell Stem Cell 16:576–577. 26046755 10.1016/j.stem.2015.05.010

[CIT0020] García-Cabezas MA , John YJ, Barbas H, Zikopoulos B (2016) Distinction of neurons, glia and endothelial cells in the cerebral cortex: an algorithm based on cytological features. Front Neuroanat 10:107. 27847469 10.3389/fnana.2016.00107 PMC5088408

[CIT0021] Gong L , Zhu T, Chen C, Xia N, Yao Y, Ding J, Xu P, Li S, Sun Z, Dong X, Shen W, Sun P, Zeng L, Xie Y, Jiang P (2022) Miconazole exerts disease-modifying effects during epilepsy by suppressing neuroinflammation via NF-κB pathway and iNOS production. Neurobiol Dis 172:105823. 35878745 10.1016/j.nbd.2022.105823

[CIT0022] Han X , Wang W, Shao F, Li N (2011) Isolation rearing alters social behaviors and monoamine neurotransmission in the medial prefrontal cortex and nucleus accumbens of adult rats. Brain Res 1385:175–181. 21338587 10.1016/j.brainres.2011.02.035

[CIT0023] Hargrove TY , Friggeri L, Wawrzak Z, Qi A, Hoekstra WJ, Schotzinger RJ, York JD, Guengerich FP, Lepesheva GI (2017) Structural analyses of *Candida albicans* sterol 14ɑ-demethylase complexed with azole drugs address the molecular basis of azole-mediated inhibition of fungal sterol biosynthesis. J Biol Chem 292:6728–6743. 28258218 10.1074/jbc.M117.778308 PMC5399120

[CIT0024] Henry JD , von Hippel W, Molenberghs P, Lee T, Sachdev PS (2016) Clinical assessment of social cognitive function in neurological disorders. Nat Rev Neurol 12:28–39. 26670297 10.1038/nrneurol.2015.229

[CIT0025] Henstridge CM , Hyman BT, Spires-Jones TL (2019) Beyond the neuron-cellular interactions early in Alzheimer disease pathogenesis. Nat Rev Neurosci 20:94–108. 30643230 10.1038/s41583-018-0113-1 PMC6545070

[CIT0026] Hsu MJ , Sheu JR, Lin CH, Shen MY, Hsu CY (2010) Mitochondrial mechanisms in amyloid beta peptide-induced cerebrovascular degeneration. Biochim Biophys Acta 1800:290–296. 19698762 10.1016/j.bbagen.2009.08.003

[CIT0027] Huang WC , Zucca A, Levy J, Page DT (2020) Social behavior is modulated by valence-encoding mPFC-amygdala sub-circuitry. Cell Rep 32:107899. 32668253 10.1016/j.celrep.2020.107899 PMC7410267

[CIT0028] Husain J , Juurlink BH (1995) Oligodendroglial precursor cell susceptibility to hypoxia is related to poor ability to cope with reactive oxygen species. Brain Res 698:86–94. 8581507 10.1016/0006-8993(95)00832-b

[CIT0029] Ju TC , Chen SD, Liu CC, Yang DI (2005) Protective effects of S-nitrosoglutathione against amyloid beta-peptide neurotoxicity. Free Radic Biol Med 38:938–949. 15749390 10.1016/j.freeradbiomed.2004.12.019

[CIT0030] Kim B (2017) Western Blot Techniques. Methods Mol Biol 1606:133–139. 28501998 10.1007/978-1-4939-6990-6_9

[CIT0031] Kingsbury L , Huang S, Wang J, Gu K, Golshani P, Wu Y E, and Hong W (2019) Correlated neural activity and encoding of behavior across brains of socially interacting animals. Cell 178:429–446.e16. 31230711 10.1016/j.cell.2019.05.022 PMC6625832

[CIT0032] Kosel F , Pelley JMS, Franklin TB (2020) Behavioural and psychological symptoms of dementia in mouse models of Alzheimer’s disease-related pathology. Neurosci Biobehav Rev 112:634–647. 32070692 10.1016/j.neubiorev.2020.02.012

[CIT0033] Kumar S , Cole R, Chiappelli F, de Vellis J (1989) Differential regulation of oligodendrocyte markers by glucocorticoids: post-transcriptional regulation of both proteolipid protein and myelin basic protein and transcriptional regulation of glycerol phosphate dehydrogenase. Proc Natl Acad Sci U S A 86:6807–6811. 2475873 10.1073/pnas.86.17.6807 PMC297935

[CIT0034] Leblanc H , Ramirez S (2020) Linking social cognition to learning and memory. J Neurosci 40:8782–8798. 33177112 10.1523/JNEUROSCI.1280-20.2020 PMC7659449

[CIT0035] Leger JM , Moulias R, Vellas B, Monfort JC, Chapuy P, Robert P, Knellesen S, Gerard D (2000) Causes and consequences of elderly’s agitated and aggressive behavior [in French]. Encephale 26:32–43. 10875060

[CIT0036] Leng F , Edison P (2021) Neuroinflammation and microglial activation in Alzheimer disease: where do we go from here? Nat Rev Neurol 17:157–172. 33318676 10.1038/s41582-020-00435-y

[CIT0037] Liu J , Dietz K, DeLoyht JM, Pedre X, Kelkar D, Kaur J, Vialou V, Lobo MK, Dietz DM, Nestler EJ, Dupree J, Casaccia P. (2012) Impaired adult myelination in the prefrontal cortex of socially isolated mice. Nat Neurosci 15:1621–1623. 23143512 10.1038/nn.3263 PMC3729624

[CIT0038] Lizak N , et al; MSBase Study Group (2017) Highly active immunomodulatory therapy ameliorates accumulation of disability in moderately advanced and advanced multiple sclerosis. J Neurol Neurosurg Psychiatry 88:196–203. 27683916 10.1136/jnnp-2016-313976

[CIT0039] Ma J , Zhang J, Hou WW, Wu XH, Liao RJ, Chen Y, Wang Z, Zhang XN, Zhang LS, Zhou YD, Chen Z, Hu WW (2015) Early treatment of minocycline alleviates white matter and cognitive impairments after chronic cerebral hypoperfusion. Sci Rep 5:12079. 26174710 10.1038/srep12079 PMC4502604

[CIT0040] Makinodan M , Rosen KM, Ito S, Corfas G (2012) A critical period for social experience-dependent oligodendrocyte maturation and myelination. Science 337:1357–1360. 22984073 10.1126/science.1220845 PMC4165613

[CIT0041] Mohs RC , Schmeidler J, Aryan M (2000) Longitudinal studies of cognitive, functional and behavioural change in patients with Alzheimer’s disease. Stat Med 19:1401–1409. 10844705 10.1002/(sici)1097-0258(20000615/30)19:11/12<1401::aid-sim432>3.0.co;2-x

[CIT0042] Najm FJ , et al (2015) Drug-based modulation of endogenous stem cells promotes functional remyelination in vivo. Nature 522:216–220. 25896324 10.1038/nature14335 PMC4528969

[CIT0043] Nasrabady SE , Rizvi B, Goldman JE, Brickman AM (2018) White matter changes in Alzheimer’s disease: a focus on myelin and oligodendrocytes. Acta Neuropathol Commun 6:22. 29499767 10.1186/s40478-018-0515-3 PMC5834839

[CIT0044] Omar R , Henley SMD, Bartlett JW, Hailstone JC, Gordon E, Sauter DA, Frost C, Scott SK, Warren JD (2011) The structural neuroanatomy of music emotion recognition: evidence from frontotemporal lobar degeneration. Neuroimage 56:1814–1821. 21385617 10.1016/j.neuroimage.2011.03.002 PMC3092986

[CIT0045] Ordóñez-Gutiérrez L , Antón M, Wandosell F (2015) Peripheral amyloid levels present gender differences associated with aging in AβPP/PS1 mice. J Alzheimers Dis 44:1063–1068. 25408213 10.3233/JAD-141158

[CIT0046] Peles E , Salzer JL (2000) Molecular domains of myelinated axons. Curr Opin Neurobiol 10:558–565. 11084317 10.1016/s0959-4388(00)00122-7

[CIT0047] Pietropaolo S , Delage P, Lebreton F, Crusio WE, Cho YH (2012) Early development of social deficits in APP and APP-PS1 mice. Neurobiol Aging 33:e17–e27. 10.1016/j.neurobiolaging.2011.09.012 22014620

[CIT0048] Rankin KP , Santos-Modesitt W, Kramer JH, Pavlic D, Beckman V, Miller BL (2008) Spontaneous social behaviors discriminate behavioral dementias from psychiatric disorders and other dementias. J Clin Psychiatry 69:60–73. 18312039 10.4088/jcp.v69n0109 PMC2735556

[CIT0049] Roth AD , Ramírez G, Alarcón R, Von Bernhardi R (2005) Oligodendrocytes damage in Alzheimer’s disease: beta amyloid toxicity and inflammation. Biol Res 38:381–387. 16579521 10.4067/s0716-97602005000400011

[CIT0050] Schmittgen TD , Livak KJ (2008) Analyzing real-time PCR data by the comparative C(T) method. Nat Protoc 3:1101–1108. 18546601 10.1038/nprot.2008.73

[CIT0051] Serrano-Pozo A , Frosch MP, Masliah E, Hyman BT (2011) Neuropathological alterations in Alzheimer disease. Cold Spring Harb Perspect Med 1:a006189. 22229116 10.1101/cshperspect.a006189 PMC3234452

[CIT0052] Song XY , Wu WF, Gabbi C, Dai YB, So M, Chaurasiya SP, Wang L, Warner M, Gustafsson JÅ (2019) Retinal and optic nerve degeneration in liver X receptor β knockout mice. Proc Natl Acad Sci U S A 116:16507–16512. 31371497 10.1073/pnas.1904719116 PMC6697819

[CIT0053] Stephenson J , Nutma E, van der Valk P, Amor S (2018) Inflammation in CNS neurodegenerative diseases. Immunology 154:204–219. 29513402 10.1111/imm.12922 PMC5980185

[CIT0054] Tomasello M , Vaish A (2013) Origins of human cooperation and morality. Annu Rev Psychol 64:231–255. 22804772 10.1146/annurev-psych-113011-143812

[CIT0055] Tsutsui M , Hirase R, Miyamura S, Nagayasu K, Nakagawa T, Mori Y, Shirakawa H, Kaneko S (2018) TRPM2 exacerbates central nervous system inflammation in experimental autoimmune encephalomyelitis by increasing production of CXCL2 chemokines. J Neurosci 38:8484–8495. 30201769 10.1523/JNEUROSCI.2203-17.2018 PMC6596171

[CIT0056] Wang J , Tanila H, Puoliväli J, Kadish I, van Groen T (2003) Gender differences in the amount and deposition of amyloidbeta in APPswe and PS1 double transgenic mice. Neurobiol Dis 14:318–327. 14678749 10.1016/j.nbd.2003.08.009

[CIT0057] Wang L , Cao M, Pu T, Huang H, Marshall C, Xiao M (2018) Enriched physical environment attenuates spatial and social memory impairments of aged socially isolated mice. Int J Neuropsychopharmacol 21:1114–1127. 30247630 10.1093/ijnp/pyy084 PMC6276026

[CIT0058] Xing B , Mack NR, Guo KM, Zhang YX, Ramirez B, Yang SS, Lin L, Wang DV, Li YC, Gao WJ (2021) A subpopulation of prefrontal cortical neurons is required for social memory. Biol Psychiatry 89:521–531. 33190846 10.1016/j.biopsych.2020.08.023 PMC7867585

[CIT0059] Xu Z , Xiao N, Chen Y, Huang H, Marshall C, Gao J, Cai Z, Wu T, Hu G, Xiao M (2015) Deletion of aquaporin-4 in APP/PS1 mice exacerbates brain Aβ accumulation and memory deficits. Mol Neurodegener 10:58. 26526066 10.1186/s13024-015-0056-1 PMC4631089

[CIT0060] Yeo IJ , Yun J, Son DJ, Han SB, Hong JT (2020) Antifungal drug miconazole ameliorated memory deficits in a mouse model of LPS-induced memory loss through targeting iNOS. Cell Death Dis 11:623. 32796824 10.1038/s41419-020-2619-5 PMC7429861

[CIT0061] Young KM , Psachoulia K, Tripathi RB, Dunn SJ, Cossell L, Attwell D, Tohyama K, Richardson WD (2013) Oligodendrocyte dynamics in the healthy adult CNS: evidence for myelin remodeling. Neuron 77:873–885. 23473318 10.1016/j.neuron.2013.01.006 PMC3842597

[CIT0062] Zhang P , Kishimoto Y, Grammatikakis I, Gottimukkala K, Cutler RG, Zhang S, Abdelmohsen K, Bohr VA, Misra Sen J, Gorospe M, Mattson MP (2019) Senolytic therapy alleviates Aβ-associated oligodendrocyte progenitor cell senescence and cognitive deficits in an Alzheimer’s disease model. Nat Neurosci 22:719–728. 30936558 10.1038/s41593-019-0372-9 PMC6605052

[CIT0063] Zhang R , Liu Y, Chen Y, Li Q, Marshall C, Wu T, Hu G, Xiao M (2020) Aquaporin 4 deletion exacerbates brain impairments in a mouse model of chronic sleep disruption. CNS Neurosci Ther 26:228–239. 31364823 10.1111/cns.13194 PMC6978250

