# Supplementary material for: Miconazole Promotes Cooperative Ability of a Mouse Model of Alzheimer Disease
Source: Int J Neuropsychopharmacol. 2022 Sep 16;25(11):951–67. doi: 10.1093/ijnp/pyac061 (PMC9670758; doi:10.1093/ijnp/pyac061)
Supplement: pyac061_suppl_Supplementary_Data [file pyac061_suppl_supplementary_data.docx]

**Supplementary Materials**

**Social interaction test**

The social interaction testing apparatus consists of three plastic compartments (40 cm × 40 cm × 30 cm), separated by two doors (8 cm × 8 cm). Each mouse was placed into the central compartment and allowed to explore the apparatus for 5 minutes of conditioning training. In the sociability test, an unfamiliar adolescent male mouse (stranger-1) from the same background strain was placed into the circular wire mesh cage (diameter: 9 cm, height: 15 cm) of either lateral compartment. The testing mouse was then placed back in the central compartment and allowed to explore all three compartments for 5 minutes. Time spent in the compartment previously containing stranger-1 relative to the one containing the empty chamber on the opposite side was determined. Social memory testing was conducted 10 minutes later. A novel adolescent male mouse of the same background strain (stranger-2) was introduced on the previously empty side, and the testing mouse was confined inside the central compartment once again. The ratio of time spent in the compartment containing stranger-2 to that in the opposite compartment containing stranger 1 was calculated (Pietropaolo et al., 2012).

**Y-maze test**

The Y maze consists of three arms, named novel arm (NA), starting arm and other arm (8 cm × 30 cm × 15 cm) with an angle of 120 degrees between each arm (Cao et al., 2017). The test contains two 5 minutes phases, including training stage and testing stage, with an interval of 2 hours. The NA was blocked by a black baffle during the first stage, but was opened during the second stage. The time spent in the NA and the numbers of entries into the NA were calculated.

**Novel object recognition test**

A novel object recognition task consists of two trials. In the first trial, mice were placed in an arena with two identical objects and allowed to explore for 5 minutes (Zhang et al., 2018). Following a two-hour delay, mice were placed back in the familiar arena where one object was replaced by a novel object, while the other object remained the same and mice were allowed to re-explore freely for 5 minutes. The discrimination index was obtained by calculating the time spent exploring the novel object/ (the time spent exploring novel object + the time spent exploring a familiar object). The above behavioral tests were performed by 2 independent experimenters who were blind to the treatment schedule.

**References**

Cao M, Pu T, Wang L, Marshall C, He H, Hu G, and Xiao M (2017) Early enriched physical environment reverses impairments of the hippocampus, but not medial prefrontal cortex, of socially-isolated mice. Brain Behav Immun 64: 232-243.

Pietropaolo S, Delage P, Lebreton F, Crusio W E, and Cho Y H (2012) Early development of social deficits in APP and APP-PS1 mice. Neurobiol Aging 33: 1002 e1017-1027.

Zhang R, Liu Y, Chen Y, Li Q, Marshall C, Wu T, Hu G, and Xiao M (2020) Aquaporin 4 deletion exacerbates brain impairments in a mouse model of chronic sleep disruption. CNS Neurosci Ther 26: 228-239.

**Supplementary Figure Legends**

**Supplementary Figure 1.** Behavior performance at the last day of training and testing phase of cooperative behavior tests of APP/PS1 mice and WT at different ages. (A-C) The drinking latency (A), number (B) and time (C) on the seventh day of the training period. (D-F) The co-drinking latency (D), number (E) and time (F) on the fifth day of the testing period. All data are represented as mean ± SEM. 2M-WT: n = 13, 2M-APP/PS1: n = 16, 5M-WT: n = 19, 5M-APP/PS1: n = 17, 8M-WT: n = 19, and 8M-APP/PS1: n = 18 in (A-C). n = 10 in each group that a pair of combinations are used, and mice in the same litter are combined with each other in (D-F). Data were analyzed by two-way ANOVA followed by Tukey's post hoc test. **P* < .05 and ***P* < .01, comparison between genotypes; ^#^*P* < .05 and ^###^*P* < .001, comparison between with different ages.

**Supplementary Figure 2.** Impairment of myelination in the mPFC of APP/PS1 mice increased with age. (A, B) Representative Western blot bands and the integral optical density analysis of MBP expression in the mPFC (n = 6). All data are represented as mean ± SEM. Data were analyzed by two-way ANOVA followed by Tukey's post hoc test. **P* < .05, comparison between genotypes; ^##^*P* < .01, comparison among different ages.

**Supplementary Figure 3.** Behavior performance at the last day of training and testing phase of cooperative behavior tests of APP/PS1 mice and WT with or without miconazole treatment. (A-C) The drinking latency (A), number (B) and time (C) on the seventh day of the training period. (D-F) The co-drinking latency (D), number (E) and time (F) on the fifth day of the testing period. All data are represented as mean ± SEM. WT-control: n = 10, WT-MIZ: n = 9, APP/PS1-control: n = 15 and APP/PS1-MIZ: n = 13 in (A-C). n = 10 in each group that a pair of combinations are used, and mice in the same litter are combined with each other in (D-F). **P* < .05 and ****P* < .001, comparison between genotypes; ^#^*P* < .05 and ^##^*P* < .01, comparison among different ages.

**Supplementary Figure 4.** Effects of miconazole treatment on MBP expression in the forebrain of 5-month-old APP/PS1 mice. (A) Immunohistochemical staining showing decreases in MBP positive myelin fibers in the mPFC of APP/PS1 mice were partially reversed by miconazole treatment. MBP immunoreactive products in the parietal association cortex (PtA) and hippocampus were not affect by the genotype or treatment. Scale bar: 50 μm. (B-D) The statistical diagram showing the MIOD of MBP expression in the mPFC (B), PtA (C) and hippocampus (D) of mice in each group (n = 6). All data are represented as mean ± SEM. Data were analyzed by two-way ANOVA followed by Tukey's post hoc test. **P* < .05, comparison between genotypes; ^#^*P* < .05, comparison between with or without MIZ treatment.

**Supplementary Figure 5.** Effects of miconazole treatment on synaptic integrity and synapses-related protein expression in the mPFC of 5-month-old APP/PS1 mice. (A) Representative electron microscopy images revealed that the profile of synapse structures in the mPFC was comparable between APP/PS1 mice and WT mice with or without miconazole treatment. Scale bar: 100 μm. (B, C) Quantified analysis of postsynaptic density and synaptic cleft width of mice in WT-CON (18 synapses), WT-MIZ (28 synapses), APP/PS1-CON (24 synapses) and APP/PS1-MIZ (18 synapses) mice (n = 6, at least 3 synapses in each mouse mPFC area). (D, E) Representative Western-blot bands and integral optical density analysis of PSD-95 and SYP expression levels in the mPFC of mice in each group (n = 6). All data are represented as mean ± SEM. Data were analyzed by two-way ANOVA followed by Tukey's post hoc test.

**Supplementary Figure 6.** Miconazole treatment did not affect expression levels of Aβ production, transport, and clearance related markers in the mPFC of 5-month-old APP/PS1 mice. (A-F) Representative Western blot bands (A) and the integral optical density analysis of ADAM10 (B), BACE1 (C), PS1 (D), LRP1 (E) and IDE (F). All data are represented as mean ± SEM. n = 6. Data were analyzed by two-way ANOVA followed by Tukey's post hoc test. ^###^*P* < .01, comparison between genotypes.

**Supplementary Table 1.** Antibodies used in the Immunohistochemistry (IHC), Immunofluorescence (IF) and Western blot (WB)

| **Antibodies** | **MW** (kDa) | | **Clone** | **Dilution** | | **Source** | **Catalog Number** |
| --- | --- | --- | --- | --- | --- | --- | --- |
| 6E10 | | NA | Mouse, monoclonal | | 1:1000 (IF) | Biolegend | SIG-39320 |
| ADAM10 | | 82 | Rabbit, polyclonal | | 1:1000 (WB) | Millipore | AB19026 |
| BACE1 | | 70 | Mouse, monoclonal | | 1:1000 (WB) | Millipore | MAB5308 |
| Erk1/2 | | 42 | Rabbit, monoclonal | | 1:1000 (WB) | Cell signaling technology | #4695 |
| GAPDH | | 37 | Rabbit, polyclonal | | 1:800 (WB) | Bio world | BS60630 |
| GFAP | |  | Mouse, monoclonal | | 1:1000 (IF) | Millipore | MAB360 |
| Iba1 | |  | Rabbit, monoclonal | | 1:1000 (IF) | Wako | 019-19741 |
| IDE | | 80 | Rabbit, polyclonal | | 1:1000 (WB) | Abcam | ab32216 |
| IL-1β | | 17 | Rabbit, polyclonal | | 1:1000 (WB) | Millipore | AB1832P |
| IL-6 | | 24 | Rabbit, polyclonal | | 1:1000 (WB) | Abcam | ab83339 |
| LRP1 | | 75 | Rabbit, polyclonal | | 1:1000 (WB) | Abcam | Ab92554 |
| MBP | | 15,17,22 | Rat, monoclonal | | 1:1000 (WB) | AdipoGen | Ab7349 |
|  | |  |  | | 1:500 (IHC) |  |  |
|  | |  |  | | 1:400 (IF) |  |  |
| NeuN | | NA | Rabbit, monoclonal | | 1:500 (IF) | Abcam | Ab177487 |
| O4 | | NA | Mouse, monoclonal | | 1:200 (IF) | R&D | MAB1326 |
| p-Erk1/2 | | 42 | Rabbit, monoclonal | | 1:1000 (WB) | Cell signaling technology | #4370 |
| PSD95 | | 85 | Rabbit, polyclonal | | 1:1000 (WB) | Abcam | ab18258 |
| PS1 | | 45 | Rabbit, polyclonal | | 1:1000 (WB) | Sigma | PRS4203 |
| SYP | | 38 | Rabbit, polyclonal | | 1:500 (WB) | Abcam | ab64581 |
| TNF-α | | 26 | Rabbit, polyclonal | | 1:1000 (WB) | Abcam | ab9739 |

ADAM10, a disintegrin and metalloproteinase domain-containing protein 10; BACE1, β-site APP-cleaving enzyme 1; Erk1/2, extracellular regulated protein kinase 1/2; GAPDH, glyceraldehyde-3-phosphate dehydrogenase; GFAP, glial fibrillary acidic protein; Iba1, ionized calcium binding adapter molecule 1; IDE, insulin-degrading enzyme; IL-1β, interleukin-1β; IL-6, interleukin-6; LRP1, low-density lipoprotein receptor-related protein 1; MBP, myelin basic protein; NeuN, neuronal nuclei; p-Erk1/2, phosphorylation-extracellular regulated protein kinase1/2; PSD95, synaptic density 95; PS1, presenilin-1; SYP, synaptophysin; TNF-α: tumor necrosis factor-α.

**Supplementary Table 2** qRT-PCR primers

| **Primers** | **Primer sequence (5'to3')** |
| --- | --- |
| Olig2-F | GGCGGTGGCTTCAAGTCAT |
| Olig2-R | CATGGCGATGTTGAGGTCG |
| Sox10-F | AGGTTGCTGAACGAAAGTGAC |
| Sox10-R | CCGAGGTTGGTACTTGTAGTCC |
| PDGFRα-F | GGAGACTCAAGTAACCTTGCAC |
| PDGFRCTC | TCAGTTCTGACGTTGCTTTCAA |
| CC1-F | GGCGTGAAATCCGAGTCCTTC |
| CC1-R | CCACCTGCAATAACTCTGCAA |
| MBP-F | GACCATCCAAGAAGACCCCAC |
| MBP-R | GCCATAATGGGTAGTTCTCGTGT |
| GAPDH-F | AGGTCGGTGTGAACGGATTTG |
| GAPDH-R | GGGGTCGTTGATGGCAACA |
