# Supplementary figures and images for: Miconazole Promotes Cooperative Ability of a Mouse Model of Alzheimer Disease
Source: Int J Neuropsychopharmacol. 2022 Sep 16;25(11):951–67. doi: 10.1093/ijnp/pyac061 (PMC9670758; doi:10.1093/ijnp/pyac061)

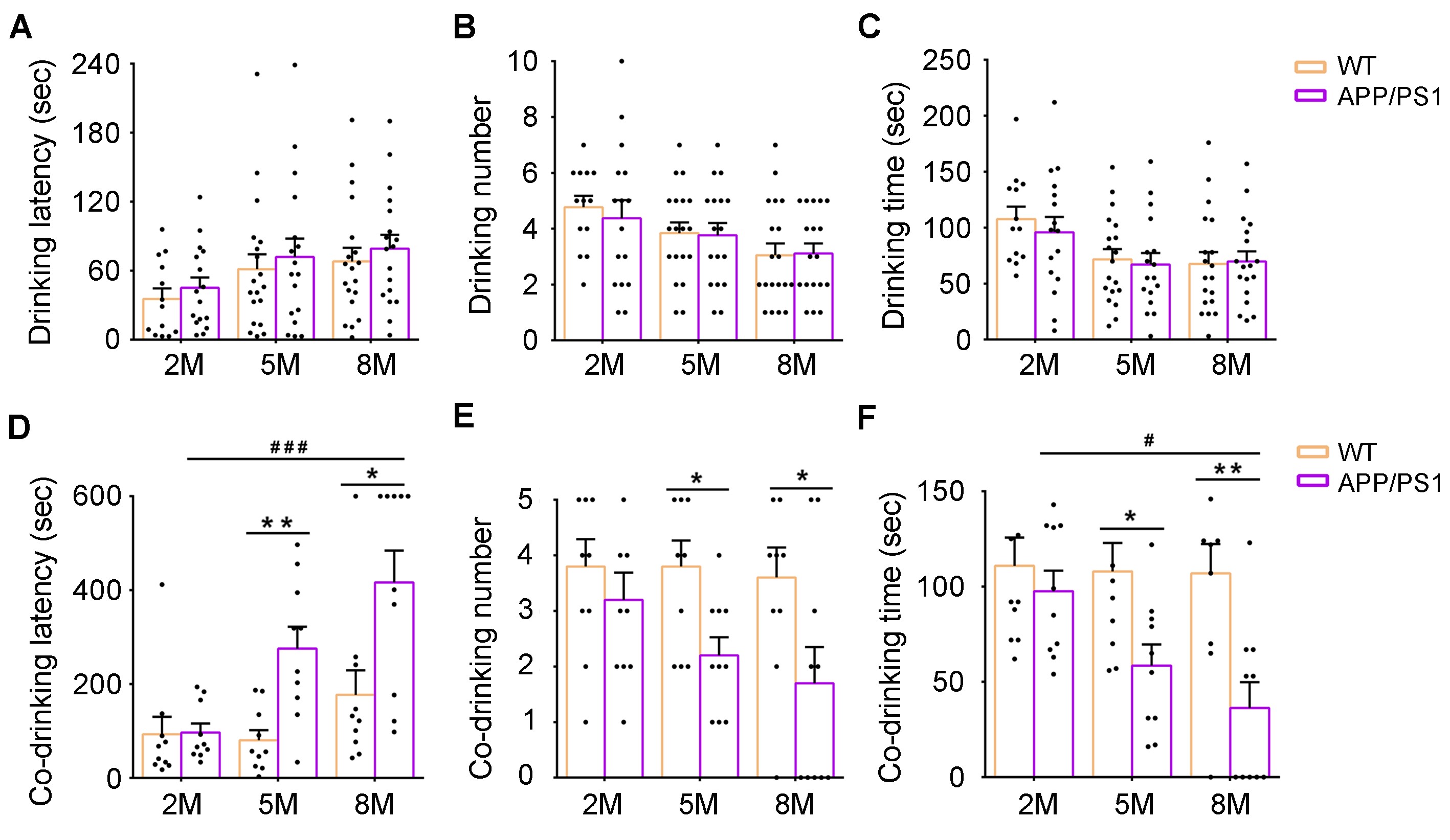

Supplement: pyac061_suppl_Supplementary_Figure_S1 [file pyac061_suppl_supplementary_figure_s1.jpeg]

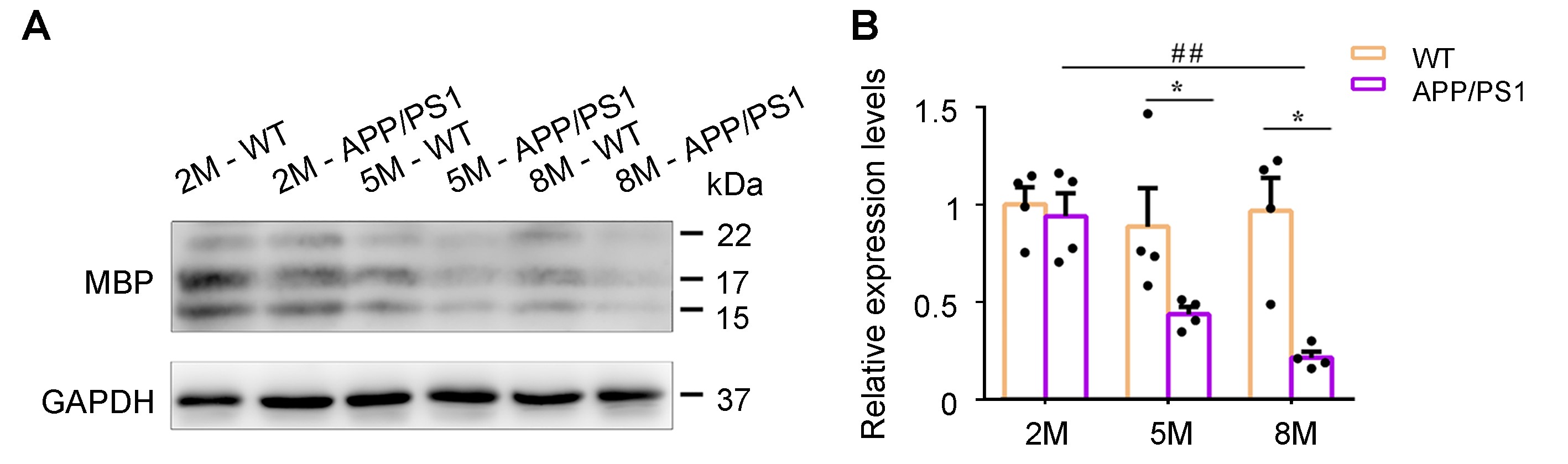

Supplement: pyac061_suppl_Supplementary_Figure_S2 [file pyac061_suppl_supplementary_figure_s2.jpeg]

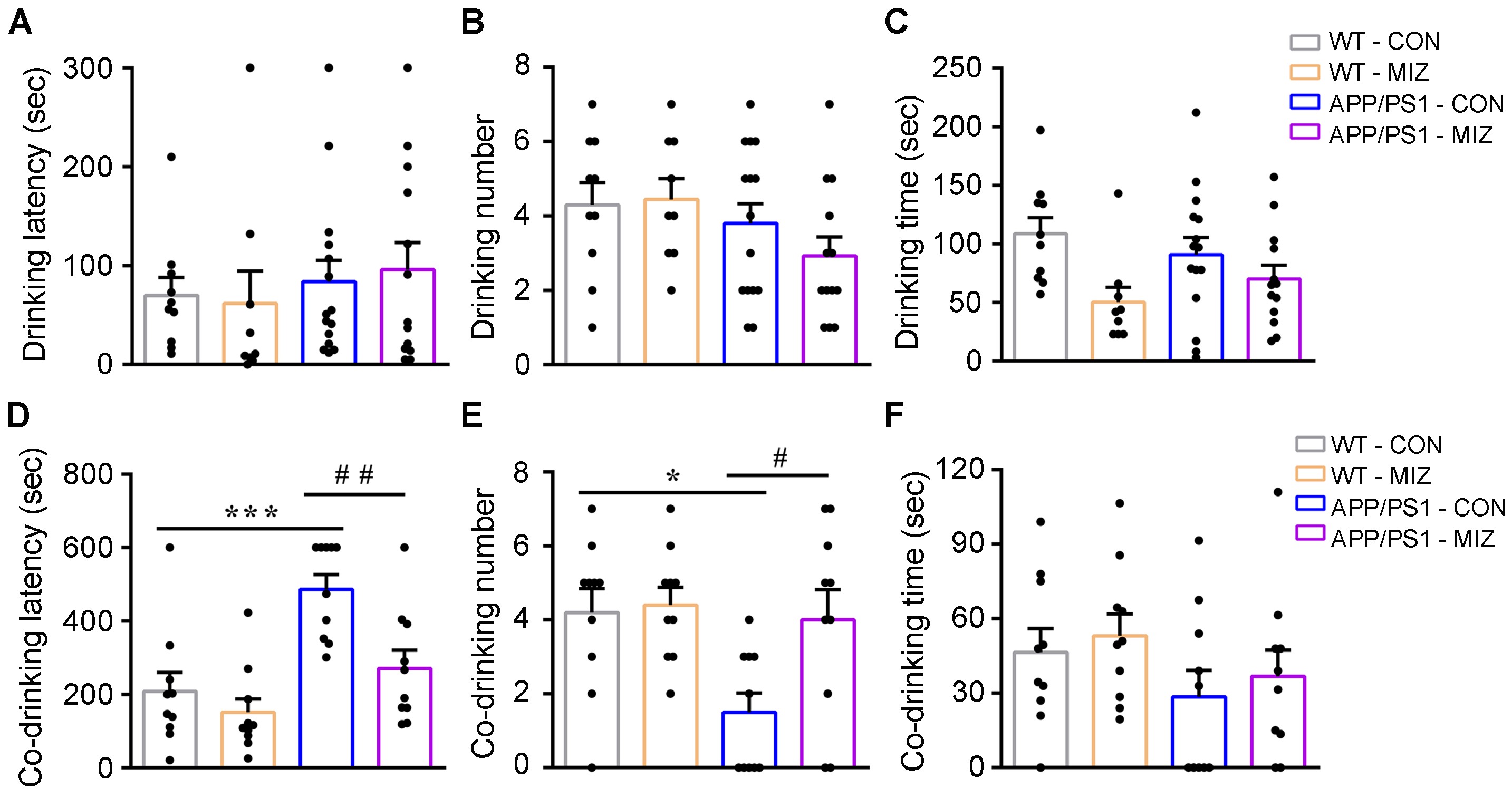

Supplement: pyac061_suppl_Supplementary_Figure_S3 [file pyac061_suppl_supplementary_figure_s3.jpeg]

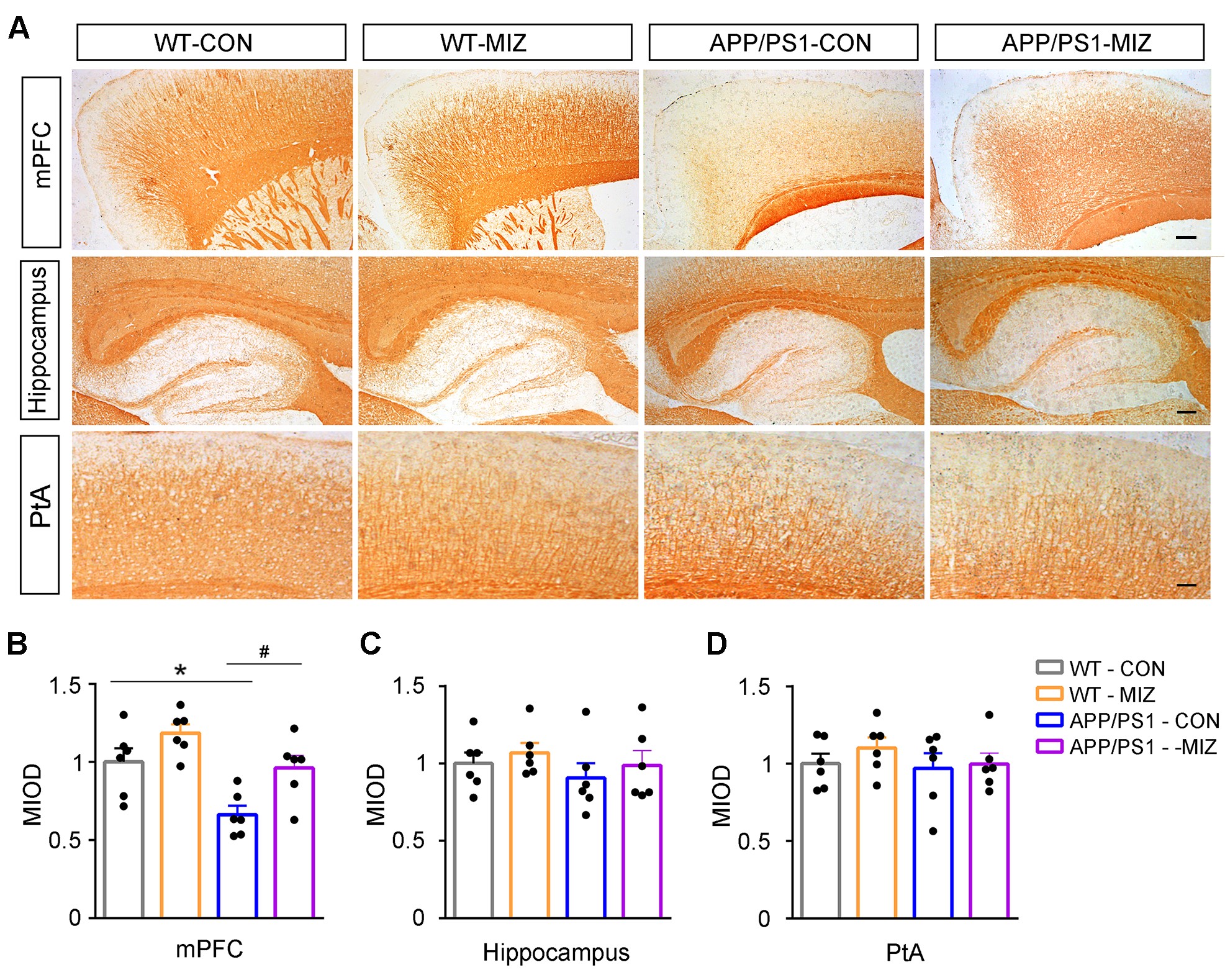

Supplement: pyac061_suppl_Supplementary_Figure_S4 [file pyac061_suppl_supplementary_figure_s4.jpeg]

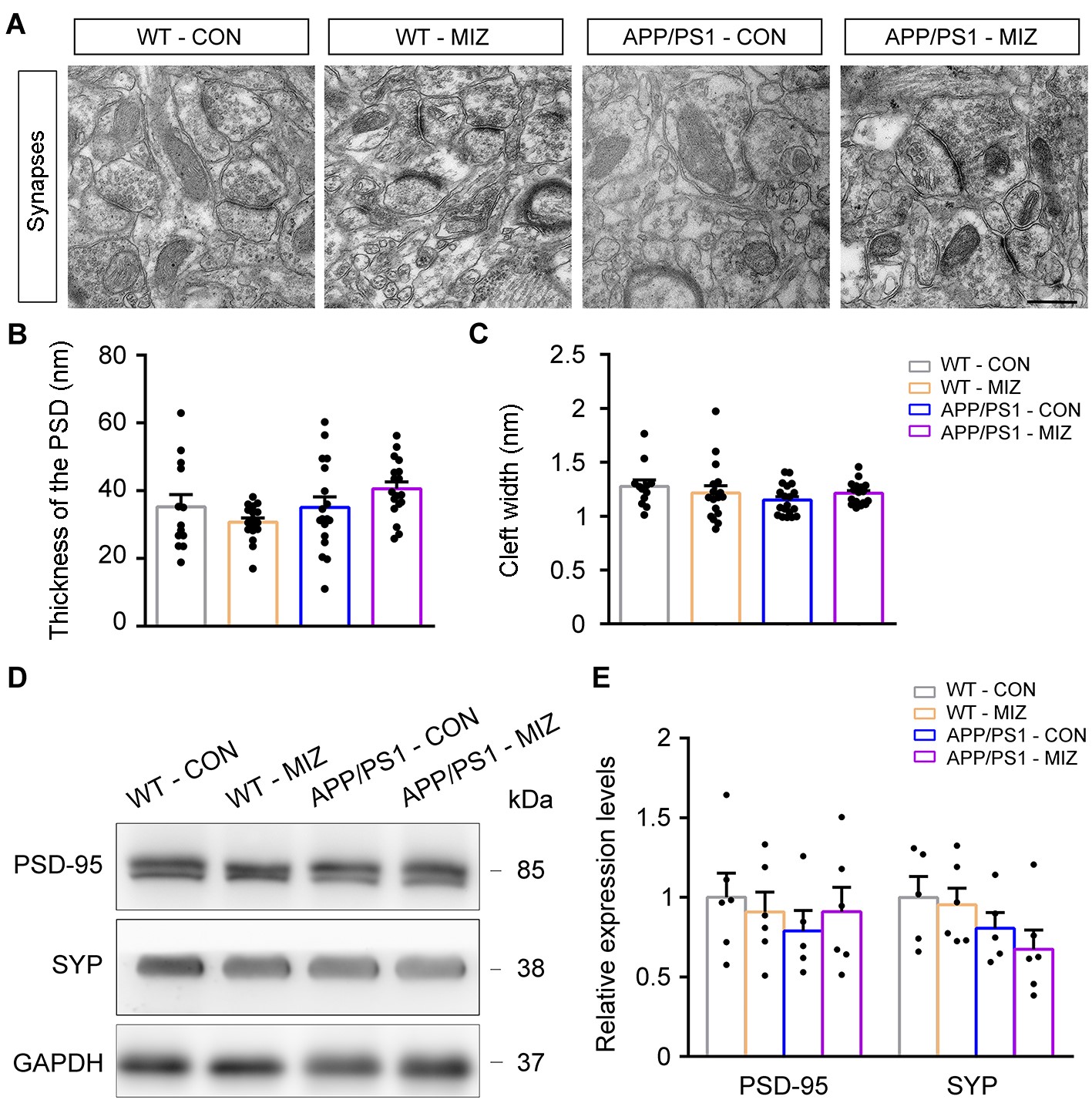

Supplement: pyac061_suppl_Supplementary_Figure_S5 [file pyac061_suppl_supplementary_figure_s5.jpeg]

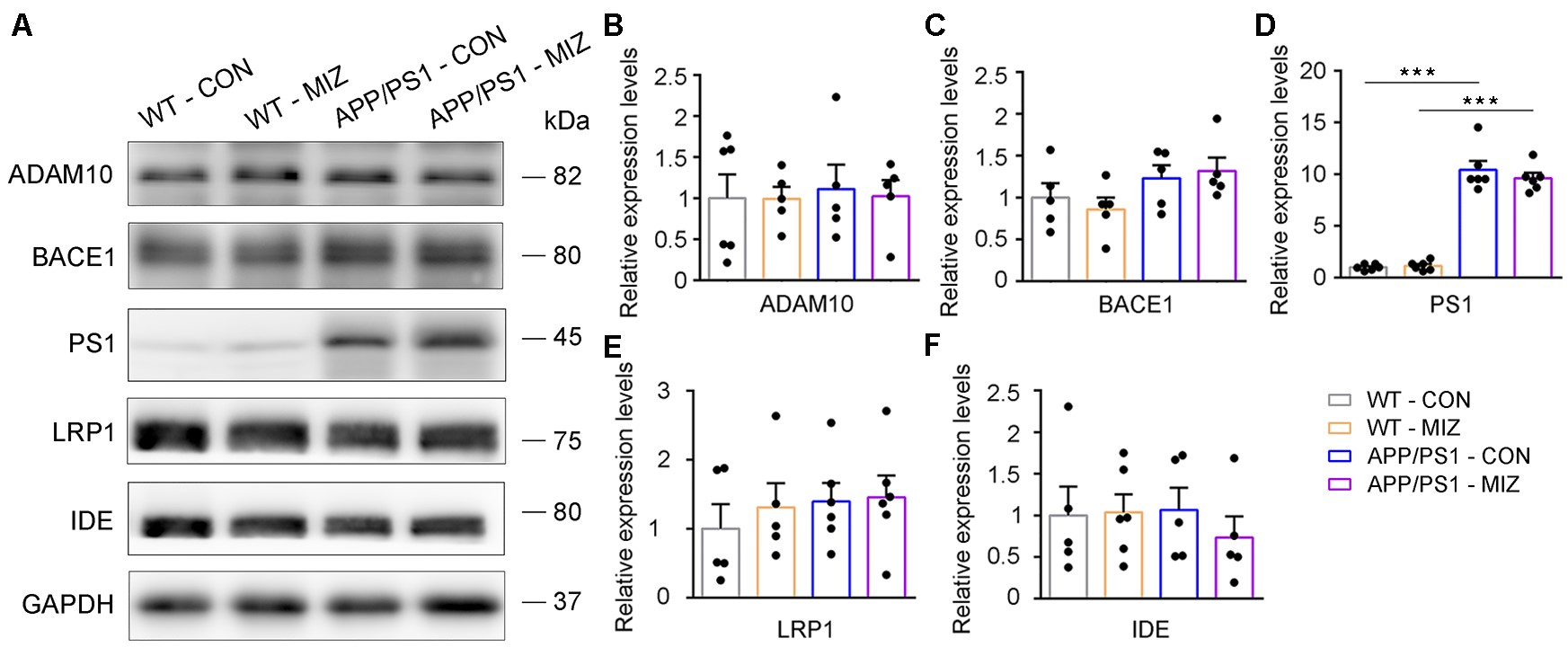

Supplement: pyac061_suppl_Supplementary_Figure_S6 [file pyac061_suppl_supplementary_figure_s6.jpeg]
