# Supplementary material for: Miconazole Promotes Cooperative Ability of a Mouse Model of Alzheimer Disease
Source: Int J Neuropsychopharmacol. 2022 Sep 16;25(11):951–67. doi: 10.1093/ijnp/pyac061 (PMC9670758; doi:10.1093/ijnp/pyac061)

**1. All uncropped bands of Figure 3F.**


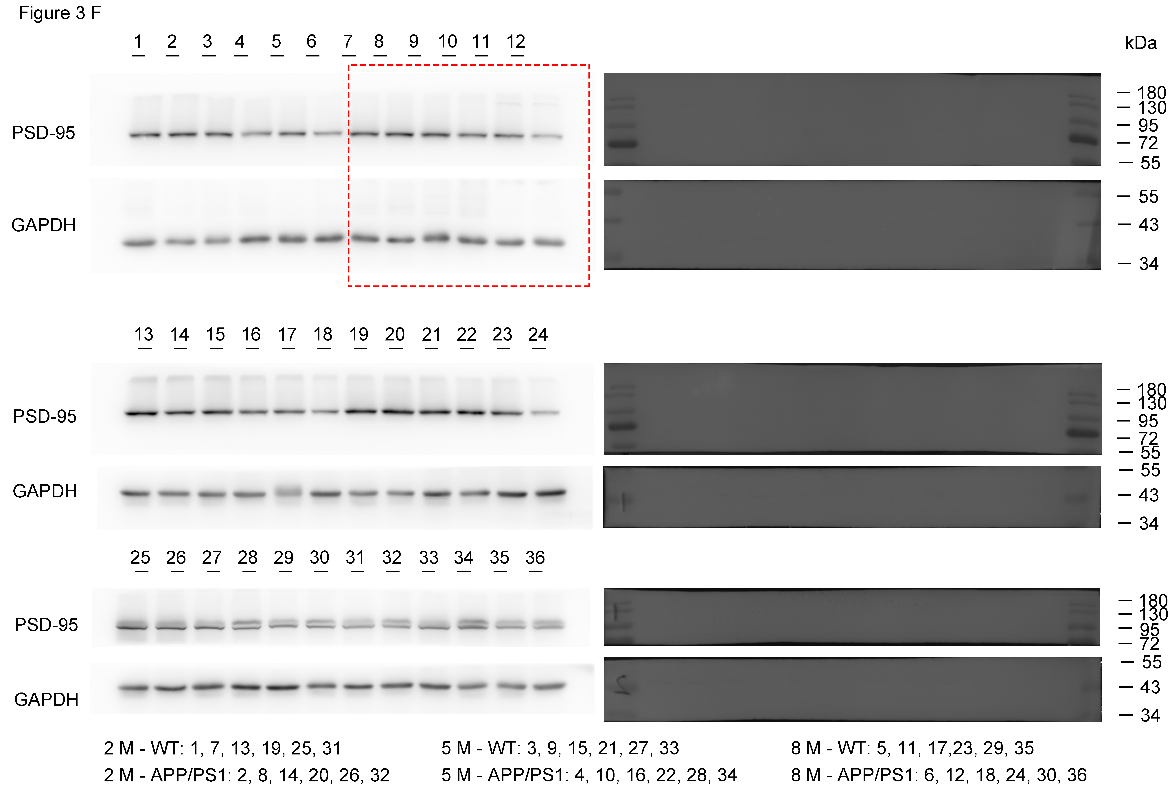


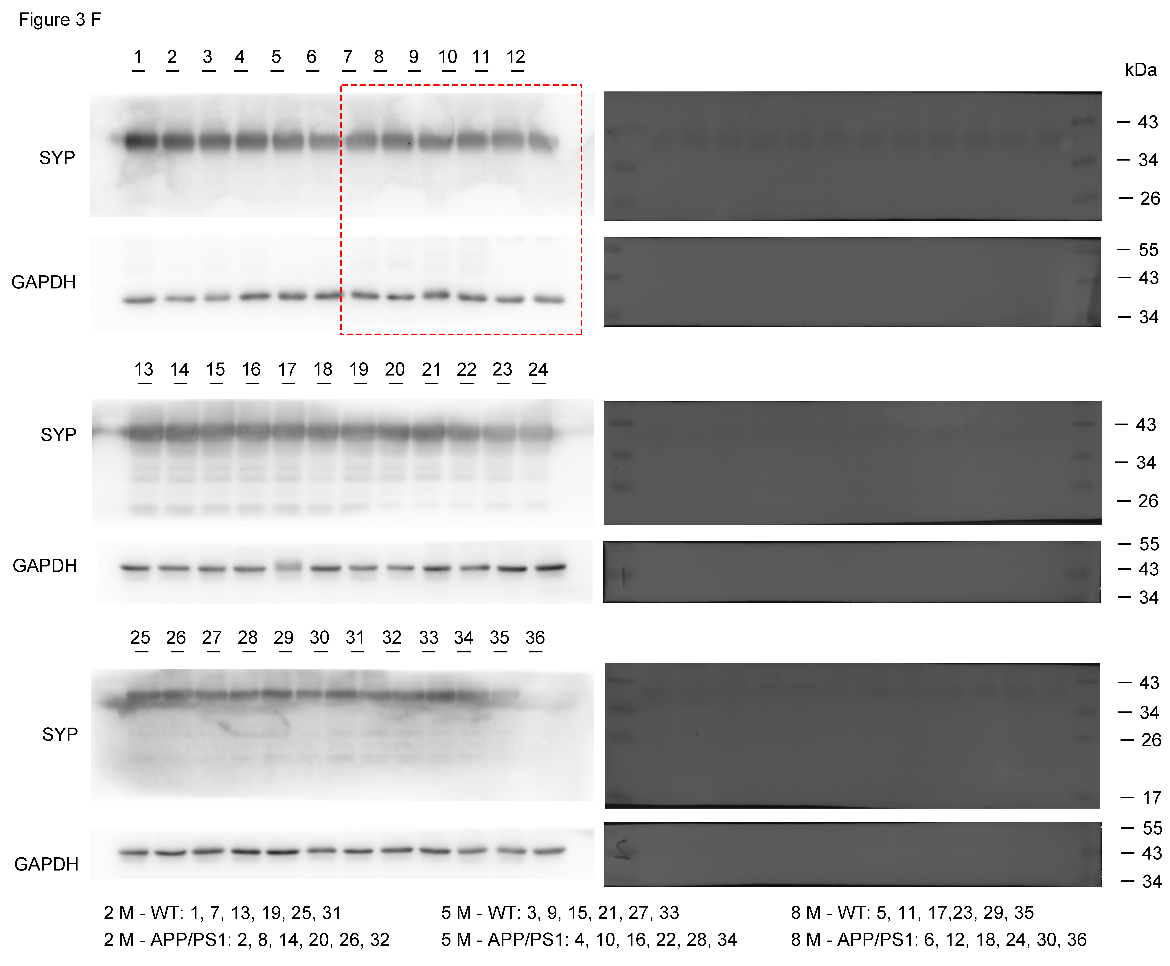


**2. All uncropped bands of Figure 6F.**


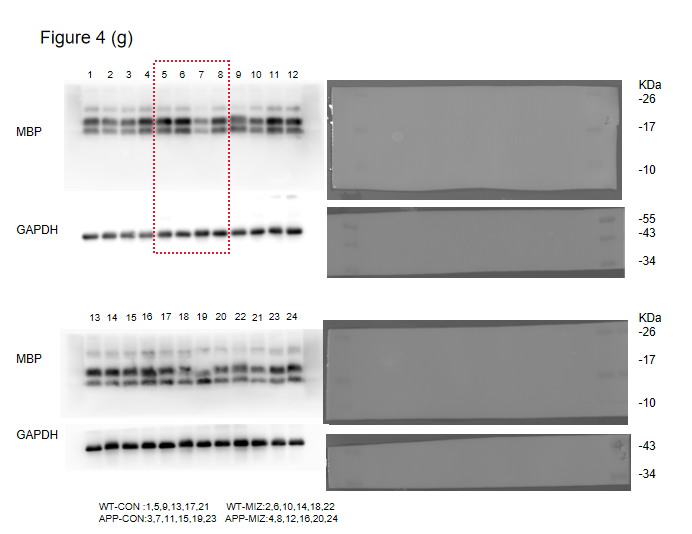


**3. All uncropped bands of Figure 7H.**


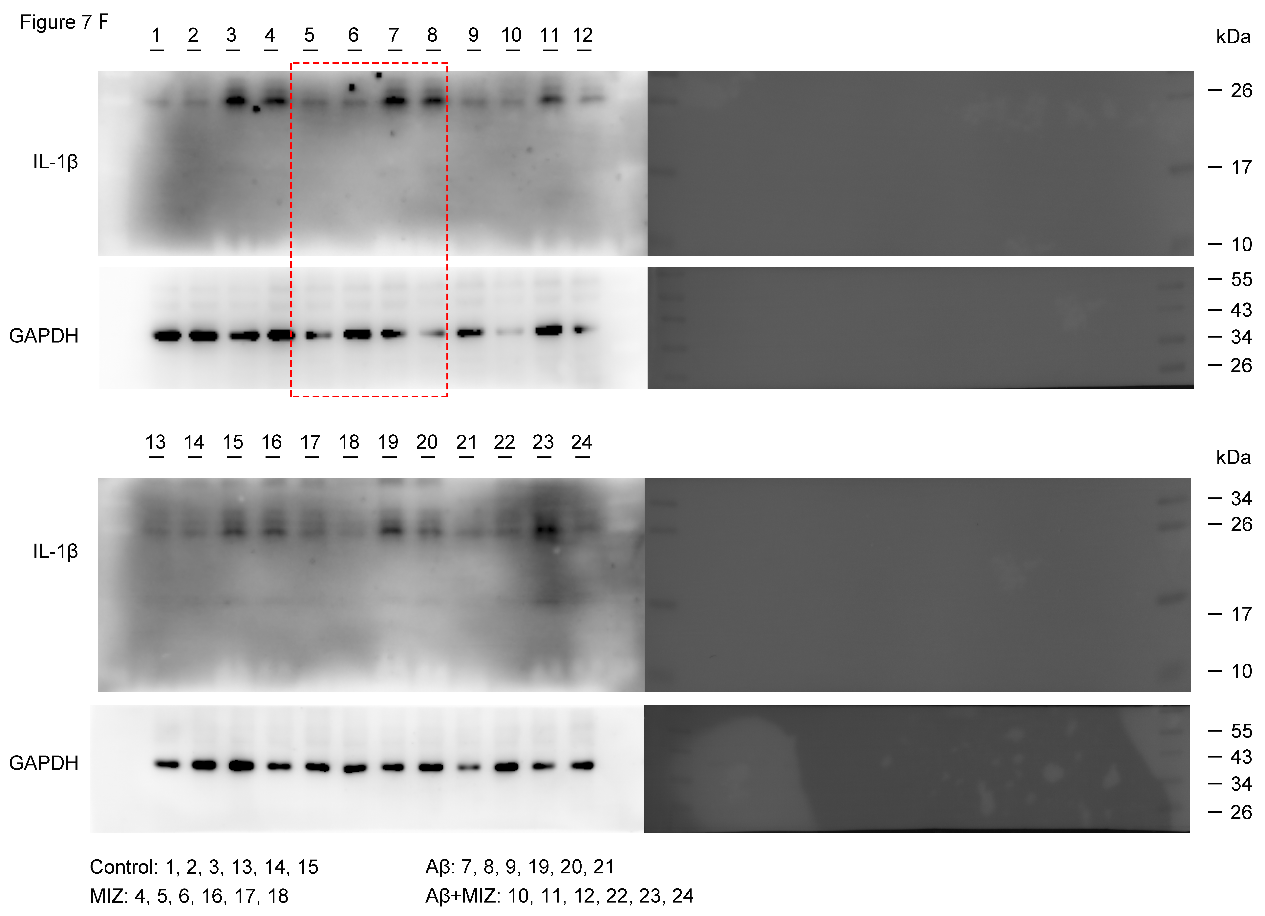


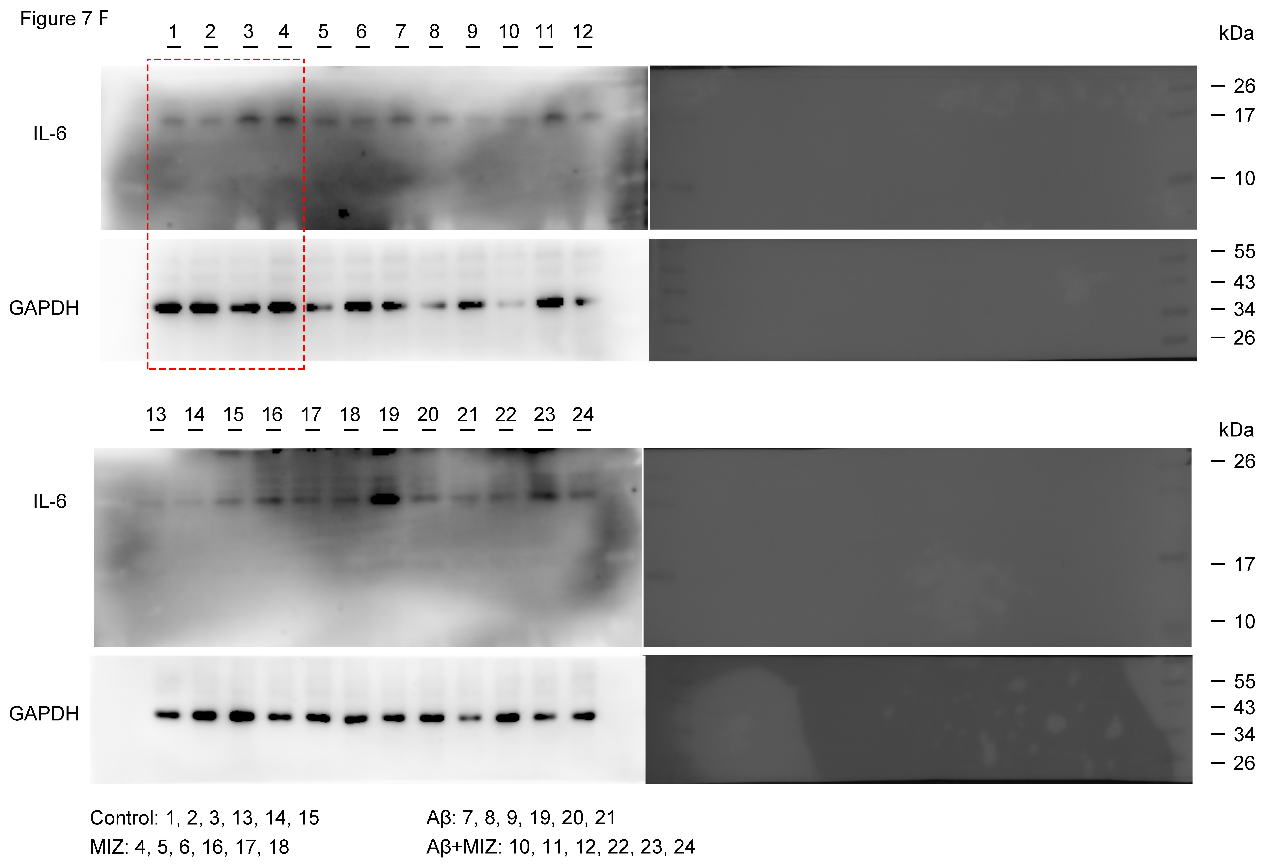


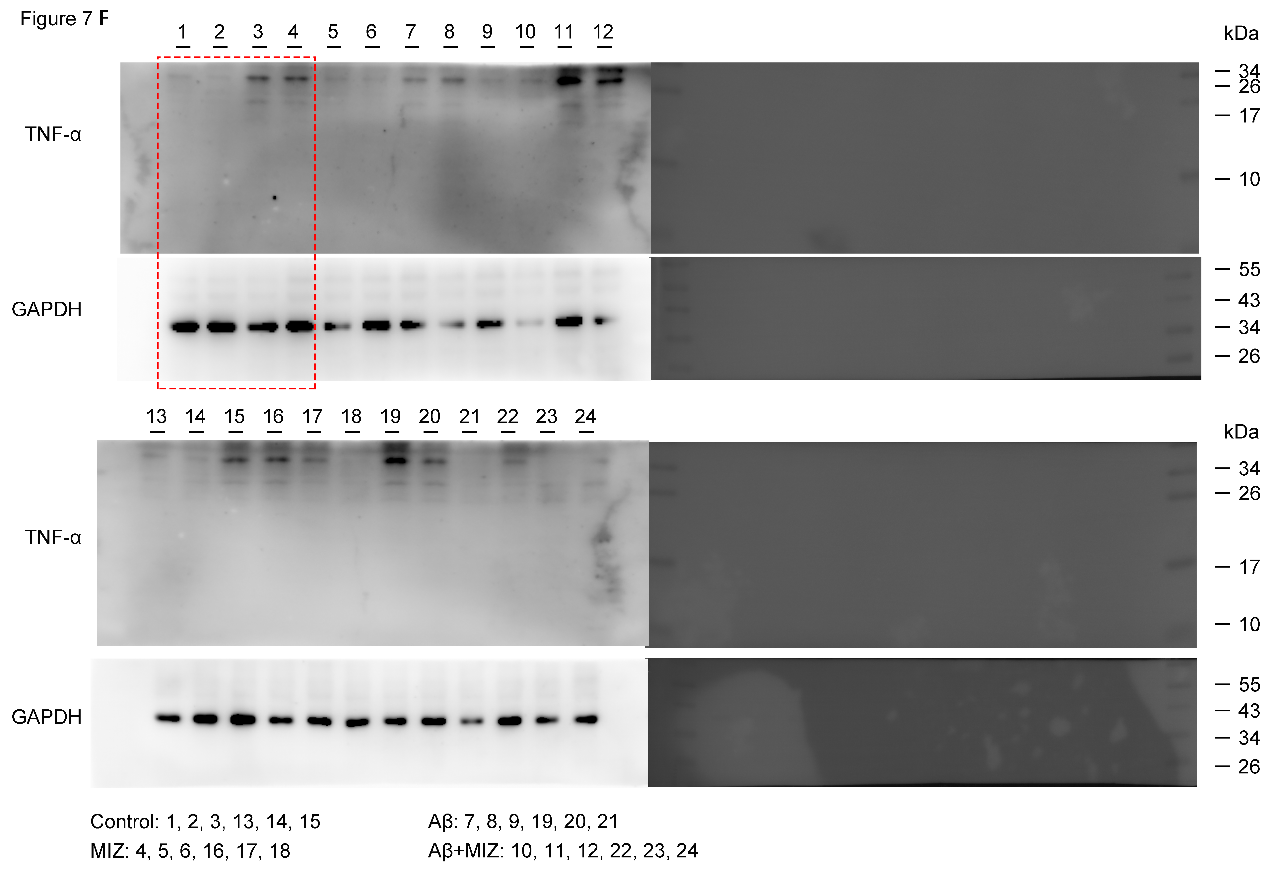


**4. All uncropped bands of Figure 8G.**


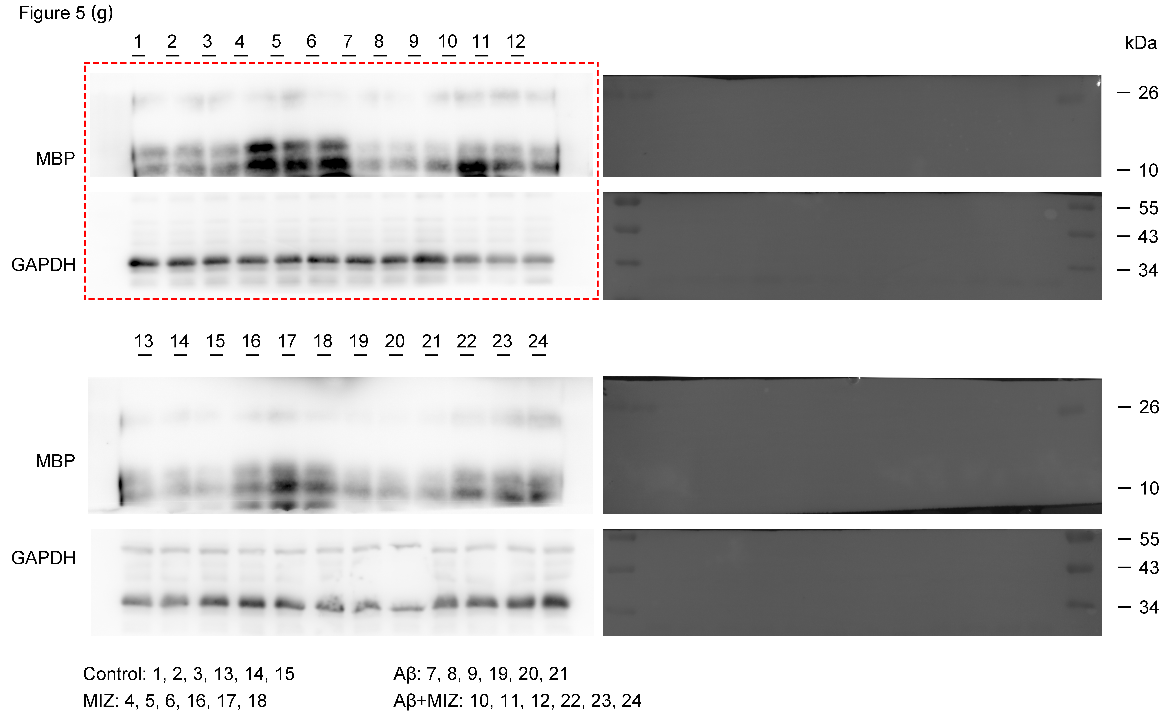


**5. All uncropped bands of Figure 8H.**


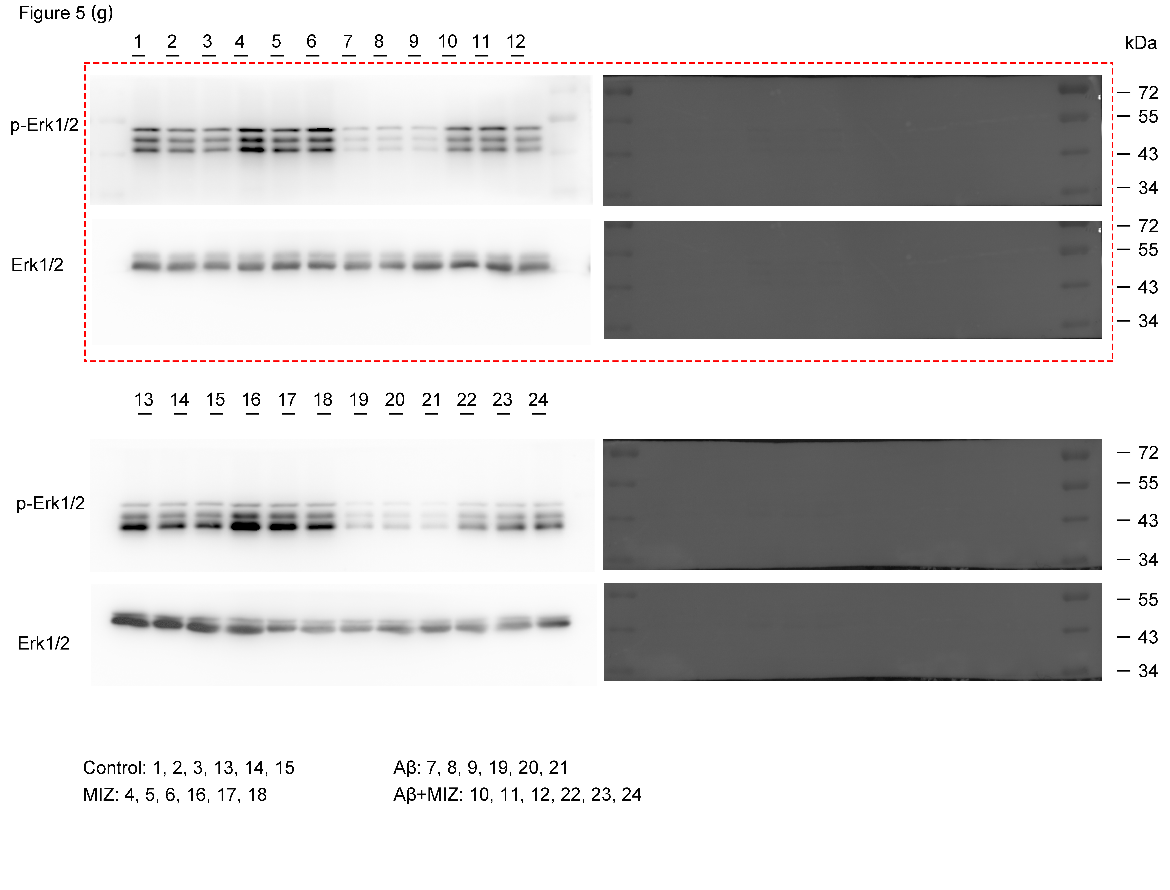


**6. All uncropped bands of Figure S2A.**


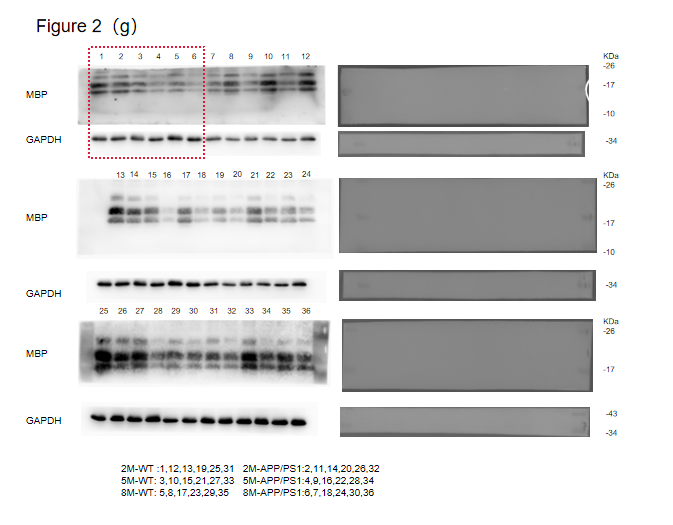


**7. All uncropped bands of Figure S6A.**


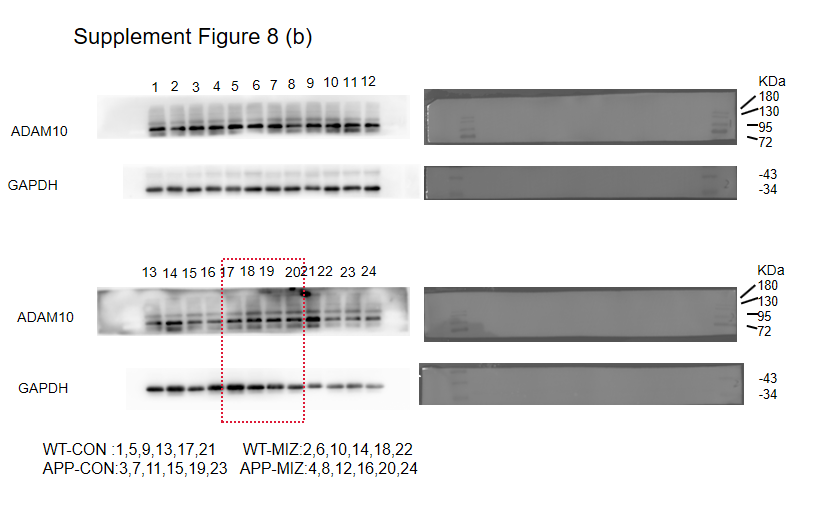


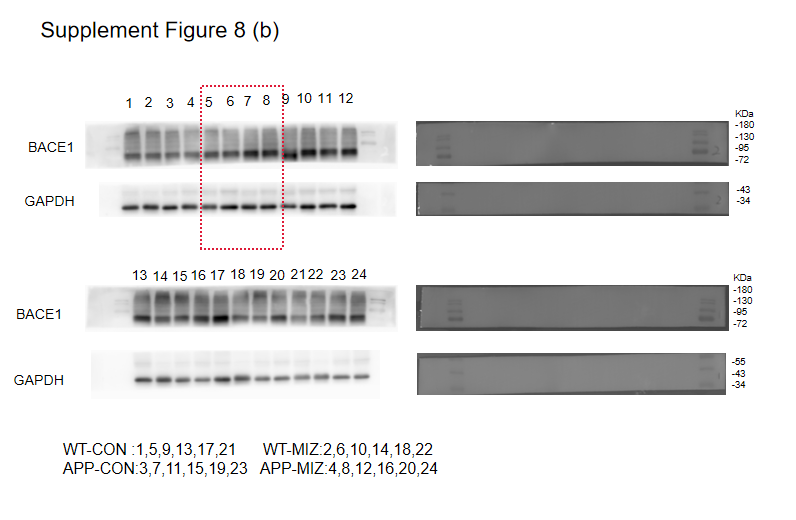


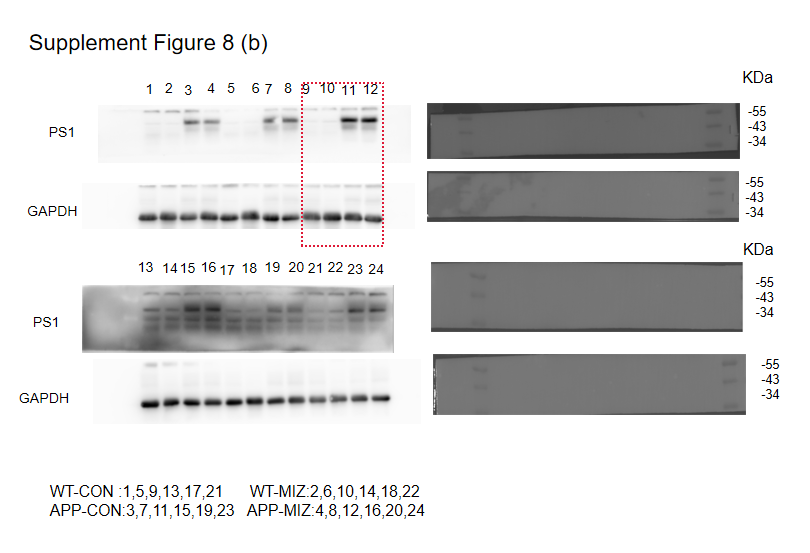


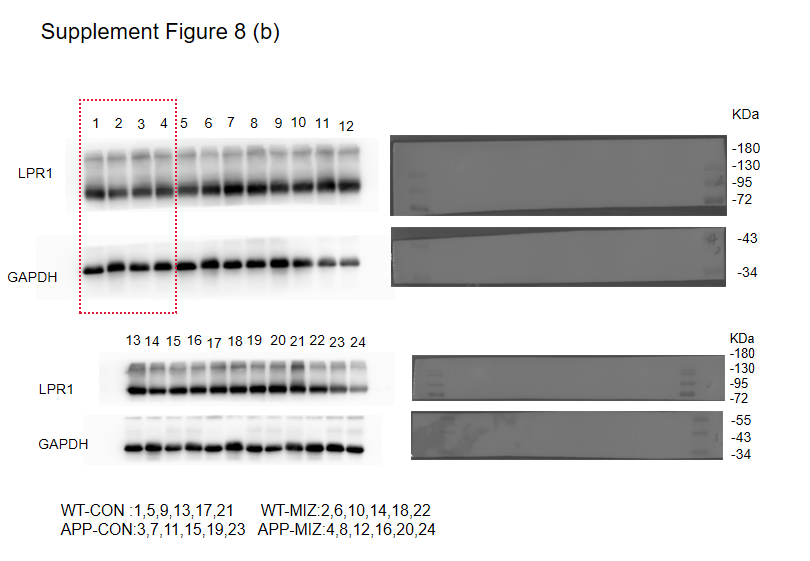


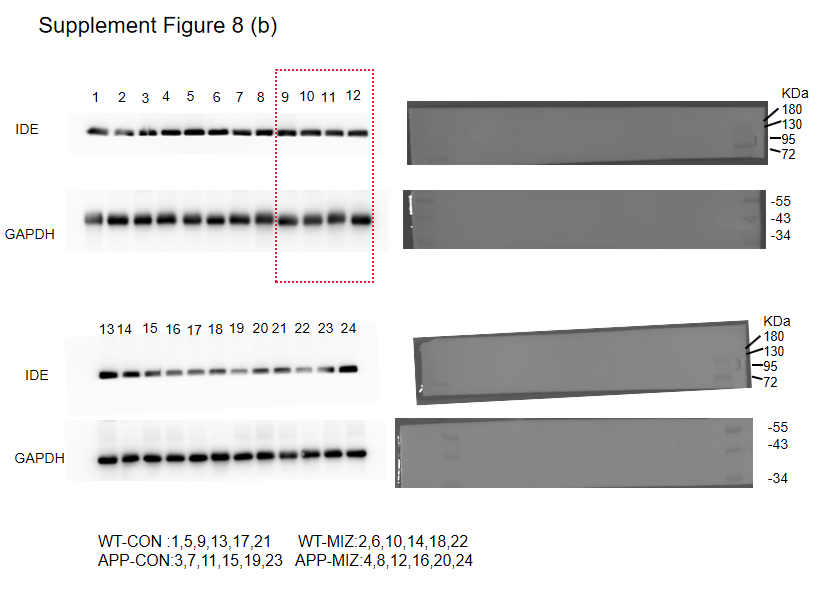

Supplement: pyac061_suppl_Supplementary_Material [file pyac061_suppl_supplementary_material.docx]
